# Dnmt1 Alleviates S1PR1‐Mediated Pyroptosis after Spinal Cord Injury through Regulating Pon3 Expression

**DOI:** 10.1002/advs.202507330

**Published:** 2025-08-30

**Authors:** Birong Peng, Haolong Lin, Mi Zhang, Wenhao Kuang, Jiaqi Zhang, Shuai Wang, Yuanfang Sun, Wenning Xu, Lixin Zhu

**Affiliations:** ^1^ Department of Spinal Surgery Orthopedic Medical Center Zhujiang Hospital Southern Medical University Guangzhou 510280 China

**Keywords:** Dnmt1, Pon3, pyroptosis, S1PR1, spinal cord injury

## Abstract

Spinal cord injury (SCI), as a severe neurological disorder, remains a formidable challenge in clinical treatment. Pyroptosis‐triggered neuroinflammation exacerbates secondary damage, neuronal death, and impairs recovery after SCI, making it a critical pathological factor. However, the exact pathophysiological mechanisms are incompletely understood. In this study, bioinformatics tools are first employed to identify key targets associated with SCI. Subsequent western blot and immunofluorescence assays reveal a time‐dependent decrease in Pon3 expression, which is predominantly localized in neuronal cells. Conversely, Dnmt1 expression shows a progressive increase following SCI. By constructing a Pon3‐overexpressing virus, it is demonstrated that Pon3 overexpression mitigates pyroptosis in rat and PC12 cells. This process promotes autophagy, significantly improving the prognosis of SCI in rats. Moreover, it enhances the survival rate of PC12 cells. mRNA sequencing and follow‐up experiments revealed that Pon3 inhibits the downstream target S1PR1, promotes autophagy, and thereby suppresses pyroptosis. Additionally, through the use of a Dnmt1 inhibitor and the knockout of the Pon3 gene, it is shown that Dnmt1 alleviates SCI‐induced pyroptosis by modulating Pon3 expression. Collectively, this work reveals that Dnmt1 alleviates S1PR1‐mediated pyroptosis following SCI via regulating Pon3 expression.

## Graphical abstract

This study systematically explores the action mechanism of Pon3 and its regulatory targets in spinal cord injury (SCI). It demonstrates that Dnmt1 regulates Pon3 expression by modulating methylation levels, thereby inhibiting S1PR1‐mediated pyroptosis and improving SCI prognosis.

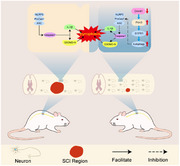

## 1 Introduction

Spinal cord injury (SCI) represents a grave neurological affliction. Mechanical injury or disease processes that damage spinal cord tissues often lead not only to sensory and motor dysfunctions below the injury level, which, in severe cases, may result in paraplegia or quadriplegia,^[^ ^1^, ^2^ ^]^ but also trigger a series of complications, such as respiratory dysfunction, urinary tract infections, pressure ulcers, and so on. These consequences significantly exacerbate the burden on patients and their families. Numerous recent studies have indicated that, globally, there are over one million patients with SCI, and the number increases by >10 000 cases annually.^[^ ^3^, ^4^ ^]^ Despite significant advancements in existing medical research and surgical care, there remains no effective treatment for the neurological deficits associated with major SCI.^[^ ^5^ ^]^

SCI can be classified into primary injury and secondary injury.^[^ ^6^, ^7^ ^]^ Inflammation and pyroptosis are significant influencing factors following secondary injury.^[^ ^8^, ^9^ ^]^ Existing evidence indicates that pyroptosis, mediated through Caspase‐1/4/5/11 activation, represents a pro‐inflammatory form of regulated cell death.^[^ ^10^ ^]^ Under physiological conditions, pyroptosis constitutes an essential part of the body's innate immune defense mechanism. It enables the body to eliminate cells infected by pathogens and initiates an inflammatory response to recruit immune cells to the site of infection.^[^ ^11^, ^12^ ^]^ However, in pathological states, excessive pyroptosis can contribute to the onset and progression of inflammatory diseases, such as vascular pathologies, progressive neurological disorders, and chronic digestive system inflammation.^[^ ^13^ ^]^ Studies have shown that autophagy can regulate axonal regeneration to alleviate nerve injury, thereby exerting neuroprotective effects against SCI. Additionally,^[^ ^14^ ^]^ research has indicated that the restoration of autophagic flux can promote recovery after SCI by inhibiting pyroptosis.^[^ ^15^, ^16^, ^17^ ^]^ Therefore, alleviating this pathological process is crucial for the treatment of SCI. Nevertheless, the regulatory mechanism of pyroptosis after SCI remains unclear and warrants further investigation.

There are three enzymes in the PON family, which are paraoxonase 1 (Pon1), paraoxonase 2 (Pon2), and paraoxonase 3 (Pon3), and they predominantly exert their functions in anti‐inflammation and antioxidation.^[^ ^18^ ^]^ Among them, Pon3 participates in diverse physiological processes encompassing proliferative regulation, senescence induction, and apoptotic/necroptotic signaling cascades.^[^ ^19^ ^]^ Research evidence indicates that Pon3 can cross the blood‐brain barrier and act on macrophages, thereby exerting its antioxidant properties.^[^ ^20^ ^]^ Additionally, other research has demonstrated that Pon3 can bind to ubiquinol (a donor of ubiquinone), inhibiting the production of superoxide, which subsequently leads to a reduction in inflammation and plays a role in atherosclerosis.^[^ ^21^ ^]^ Moreover, evidence has indicated that Pon3 can alleviate the progression of cancer by suppressing cell proliferation.^[^ ^22^ ^]^ Studies have shown that loss of Pon3 function may contribute to the development of Alzheimer's disease. Additionally,^[^ ^23^ ^]^ Pon3 may help maintain the integrity of neural myelin sheaths and protect nerves from neurotoxic effects. Furthermore,^[^ ^24^ ^]^ the loss of Pon3's antioxidant function leads to lipid peroxidation that damages neurons, ultimately exacerbating amyotrophic lateral sclerosis. However, no research has delved into the role of Pon3 in SCI. Our preliminary study has revealed that Pon3 expression significantly increases 1 day after SCI, and it is predominantly expressed in neuronal cells. This temporal and cellular‐specific expression pattern strongly suggests Pon3's potential involvement in the pathophysiological cascade following SCI. Given these findings, systematic investigation into the functional significance and molecular mechanisms of Pon3 in SCI is critically warranted.

As a key epigenetic modulator, DNA methylation dynamically controls transcriptional activity and cellular specialization through reversible covalent modifications of genomic DNA.^[^ ^25^ ^]^ Research evidence indicates that DNA methyltransferase 1 (Dnmt1) plays a crucial role in the pathogenesis, clinical diagnosis, and treatment of various diseases, and have revealed its core regulatory function in maintaining cellular homeostasis.^[^ ^26^ ^]^ Moreover, other investigations have suggested that Dnmt1 is involved in the regulation of multiple fundamental biological processes, including genomic hypomethylation, genomic stability maintenance, and cell cycle progression.^[^ ^27^, ^28^ ^]^ Abnormalities in its function can lead to pathological changes such as cytotoxic responses and abnormal cell differentiation. Additionally,^[^ ^29^ ^]^ DNA methylation is involved in pathological aspects such as axonal regeneration and inflammation following SCI.^[^ ^30^ ^]^ Studies have demonstrated that specific molecules associated with axonal regeneration after SCI are influenced by DNA methylation levels. Furthermore,^[^ ^31^ ^]^ DNA methylation regulates the level of colony‐stimulating factor‐1 (CSF1), thereby inducing spinal neuroinflammation. Collectively, these findings indicate that DNA methylation plays a critical role in the pathological process of SCI. However, the mechanism by which Dnmt1 is associated with pyroptosis following SCI remains elusive. Therefore, this study aims to address three key questions: 1) whether Pon3 serves as a critical modulator in SCI progression; 2) the mechanistic relationship between Pon3 and pyroptosis in the SCI pathological cascade; and 3) whether Dnmt1‐mediated epigenetic regulation governs Pon3 expression and contributes to functional recovery after SCI. Based on our preliminary studies, we propose a hypothesis: Pon3 affects the balance between pyroptosis and autophagy after SCI through epigenetic regulation mediated by Dnmt1.

## 2 Results

### 2.1 Temporal Changes and Spatial Localization of Pon3 Following SCI

**Figure 1:**
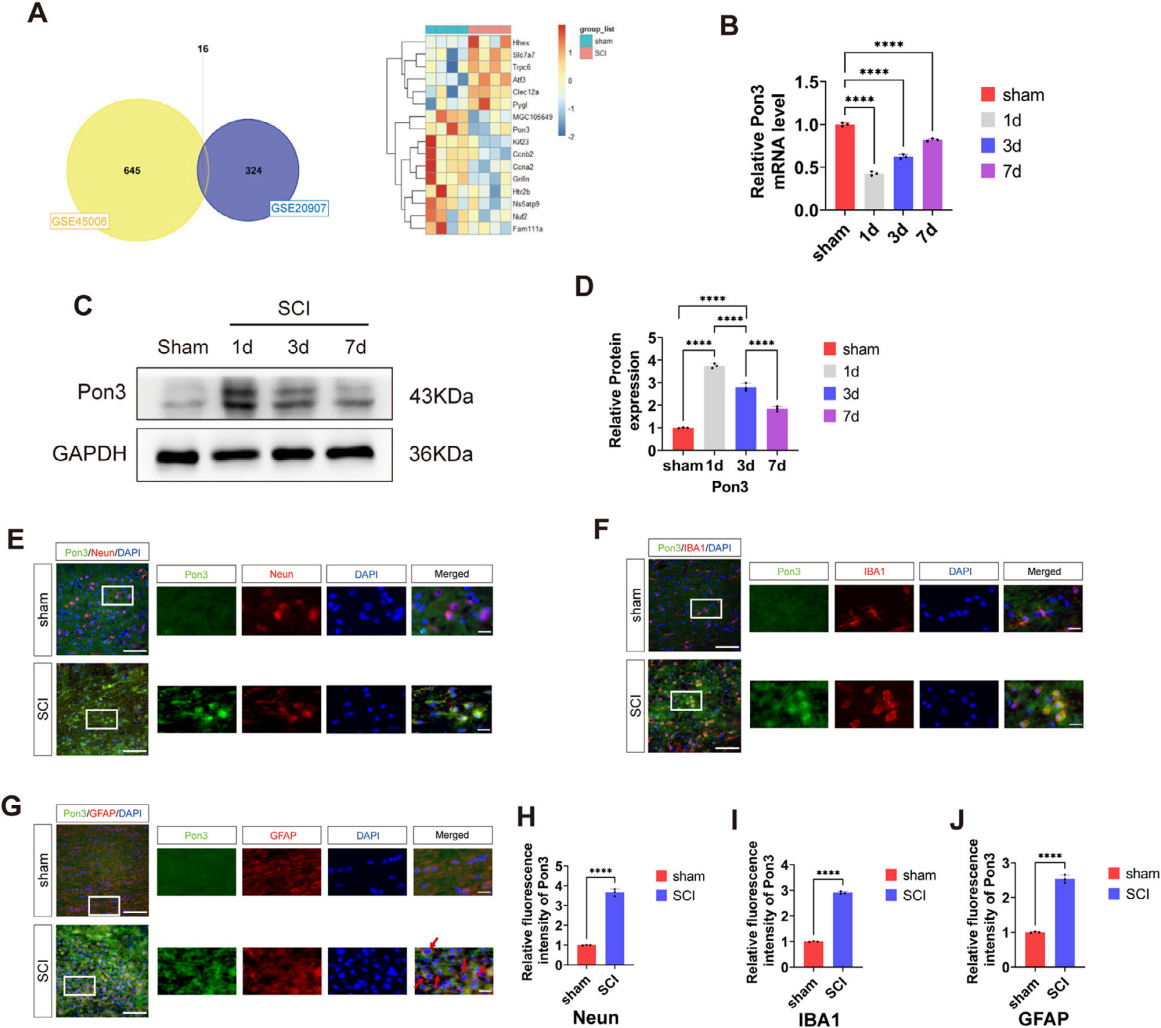
Temporal Changes and Spatial Localization of Pon3 Following SCI. A) Venn diagram and heatmap display the overlapping differentially expressed genes (DEGs) between the two datasets. B) qRT‐PCR detection of Pon3 expression levels after SCI (n = 3). C) Western blot analysis of Pon3 protein expression levels in the spinal cord at 1, 3, and 7 days after SCI. D) Quantification of Pon3 protein levels after SCI (n = 3). E–G) Double immunofluorescence staining of Pon3 (green) with Neun(red), IBA1(red), or GFAP (red) in the spinal cord at 3 days after SCI. Images were captured using confocal microscopy (Scale bars: 100 µm in main panels; 20 µm in enlarged insets). H–J) Quantitative analysis of Pon3 relative fluorescence intensity (n = 3). Data are presented as mean ± SEM. Significance was determined by two‐tailed unpaired t‐tests or one‐way ANOVA, followed by Tukey's multiple comparisons test. ****p < 0.0001.

First, through bioinformatics analysis, we screened out differentially expressed genes from two databases (**Figure** **1**A). We found that the mRNA level of Pon3 decreased after SCI (Figure 1B). To validate this result, we established a SCI model (Figure S1A,B, Supporting Information). We observed obvious congestion and edema in the spinal cord at the T9 level of the rats, and the rats developed paralysis of their hind limbs, which indicated the successful establishment of the SCI model. Subsequently, Western blot analysis revealed that the Pon3 protein reached its peak on the 1st day after injury and then gradually declined on the 3rd and 7th days (Figure 1C,D). Meanwhile, the immunofluorescence results revealed that Pon3 was expressed in neurons, microglia, and astrocytes. Notably, the level of Pon3 variation is the highest in neuronal cells (Figure 1E–J). Therefore, these results suggest that Pon3 is involved in the pathophysiological processes following SCI. Subsequently, to elucidate its functional role, we carried out the following studies.

### 2.2 Overexpression of Pon3 Improves the Functional Recovery of SCI in Rats

**Figure 2:**
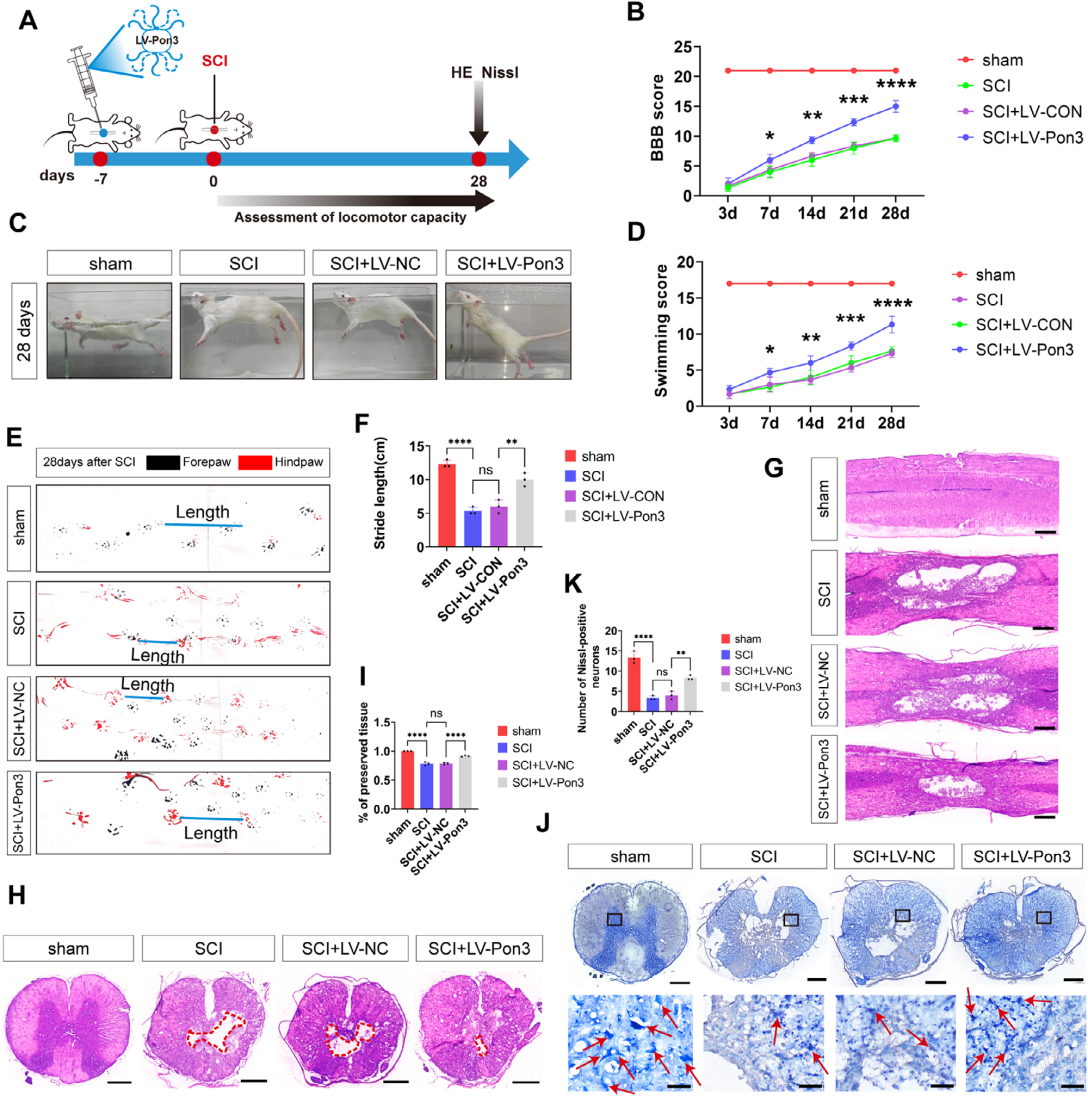
Overexpression of Pon3 Improves the Functional Recovery of SCI in Rats. A) Experimental timeline: Rats received intraspinal injection of Pon3‐overexpressing virus 7 days before SCI, followed by behavioral tests and histological analysis. B) Quantitative analysis of BBB scores at different time points after SCI (n = 3). C) Representative images of swimming tests at 28 days after SCI. D) Quantitative analysis of swimming tests at different time points after SCI (n = 3). E) Representative footprint images at 28 days after SCI. F) Quantitative analysis of footprint tests at different time points after SCI (n = 3). G–I) Sagittal and transverse HE staining images at day 28 after SCI, and quantitative analysis of the percentage of relatively preserved spinal cord tissue (n = 3; scale bar: 500 µm). J,K) Nissl staining images of spinal cord transverse sections at day 28 after SCI, and quantitative analysis of neurons (n = 3; Scale bars: 500 µm in main panels; 50 µm in enlarged insets). Data are presented as mean ± SEM. Significance was determined by one‐way ANOVA followed by Tukey's multiple comparisons test. ns: p > 0.05; * p < 0.05; ** p < 0.01; *** p <0.001; ****p <0.0001.

To explore the role of Pon3 in the aftermath of SCI, we conducted motor function assessments and histological analyses on rats (**Figure** **2**A). First, the results of Western blot and immunofluorescence demonstrated that the Pon3 gene was successfully overexpressed in rats (Figure S2A–C, Supporting Information). Subsequently, the Basso, Beattie, and Bresnahan (BBB) scoring and swimming experiments revealed that, on the 7th day after SCI, the overexpression of Pon3 significantly restored the motor function and coordination of the rats' hind limbs (Figure 2B–D). Moreover, the results of the gait analysis experiment on the 28th day after SCI further confirmed the neuroprotective effect of Pon3, compared with the SCI group and the SCI + LV‐CON group, the stride length and motor coordination in the Pon3 overexpression group were significantly improved (Figure 2E,F). Furthermore, histological analyses using hematoxylin‐eosin (HE) and Nissl staining revealed that the SCI+LV‐Pon3 group exhibited a significantly reduced lesion area and increased number of surviving neurons compared to both the SCI group and SCI+LV‐CON group (Figure 2G–K). Collectively, these findings demonstrate that Pon3 overexpression confers significant neuroprotective effects and improves functional recovery in SCI rats.

### 2.3 Overexpression of Pon3 Attenuates Pyroptosis and Promotes Autophagy after SCI

**Figure 3:**
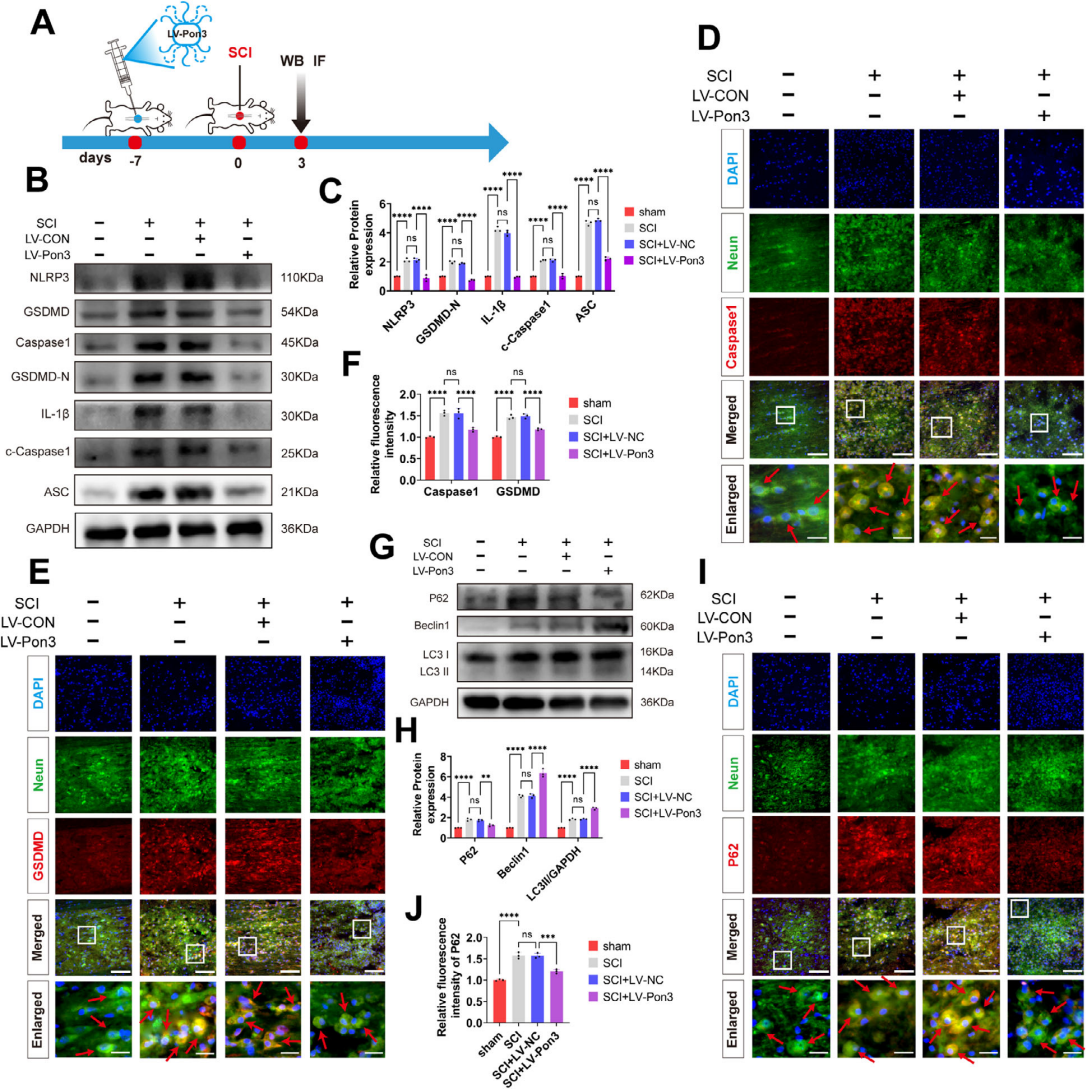
Overexpression of Pon3 Attenuates Pyroptosis and Promotes Autophagy after SCI. A) Experimental timeline: Intraspinal injection of Pon3‐overexpressing virus was performed 7 days before SCI, followed by Western blot and immunofluorescence analyses at 3 days after injury. B‐C) Western blot analysis of pyroptosis‐related proteins in spinal cord tissue at 3 days after SCI and their quantitative analysis (n = 3). D–F) Double immunofluorescence staining of Neun (green) with Caspase1 (red) or GSDMD (red) at 3 days after SCI, with quantitative analysis of relative fluorescence intensity (n = 3; scale bar: 100 µm in main panels; 20 µm in enlarged insets). G,H) Western blot analysis of autophagy‐related proteins and their quantitative analysis at 3 days after SCI (n = 3). I,J) Double immunofluorescence staining of Neun (green) with P62 (red) and quantitative analysis (n = 3; scale bar: 100 µm in main panels; 20 µm in enlarged insets). Data are presented as mean ± SEM. Significance was determined by one‐way or two‐way ANOVA, followed by Tukey's multiple comparisons test. ns: p > 0.05; ** p < 0.01; ****p <0.0001.

To investigate the mechanism of Pon3‐mediated neuroprotection in SCI, we evaluated the effects of Pon3 on the pyroptosis and autophagy pathways through Western blot and immunofluorescence analyses (**Figure** **3**A).^[^ ^32^ ^]^ Studies have shown that pyroptosis‐related proteins peak on day 3 after SCI. Therefore, we constructed SD rat SCI models at different time points and found through Western blot that pyroptosis‐related proteins gradually increased after SCI, peaked on day 3, and then gradually decreased (Figure S3A,B, Supporting Information). Therefore, we chose day 3 for Western blot and immunofluorescence experiments. Following SCI, the Western blot results revealed that the levels of pyroptosis‐related biomarkers such as NLRP3, GSDMD, Caspase1, IL‐1β, and ASC increased significantly. In contrast, overexpression of Pon3 led to a remarkable reduction in these levels (Figure 3B,C). Similarly, the immunofluorescence results demonstrated that after SCI, the expressions of GSDMD and Caspase1 in neuronal cells increased substantially, while overexpression of Pon3 could significantly decrease their levels (Figure 3D–F). Subsequently, the Western blot results showed that SCI promoted the accumulation of the autophagy substrate P62 and a notable increase in the expressions of autophagy‐related biomarkers such as Beclin1 and LC3II. However, overexpression of Pon3 could significantly decrease the level of P62 and further elevate the levels of Beclin1 and LC3II (Figure 3G,H). Meanwhile, the immunofluorescence results indicated that after SCI the level of P62 in neurons increased remarkably, and overexpression of Pon3 could significantly reduce this level (Figure 3I,J). Collectively, these results suggest that overexpression of Pon3 can alleviate pyroptosis and promote autophagy following SCI.

### 2.4 Overexpression of Pon3 Attenuates TBHP‐Induced Pyroptosis and Promotes Autophagy in PC12 Cells

**Figure 4:**
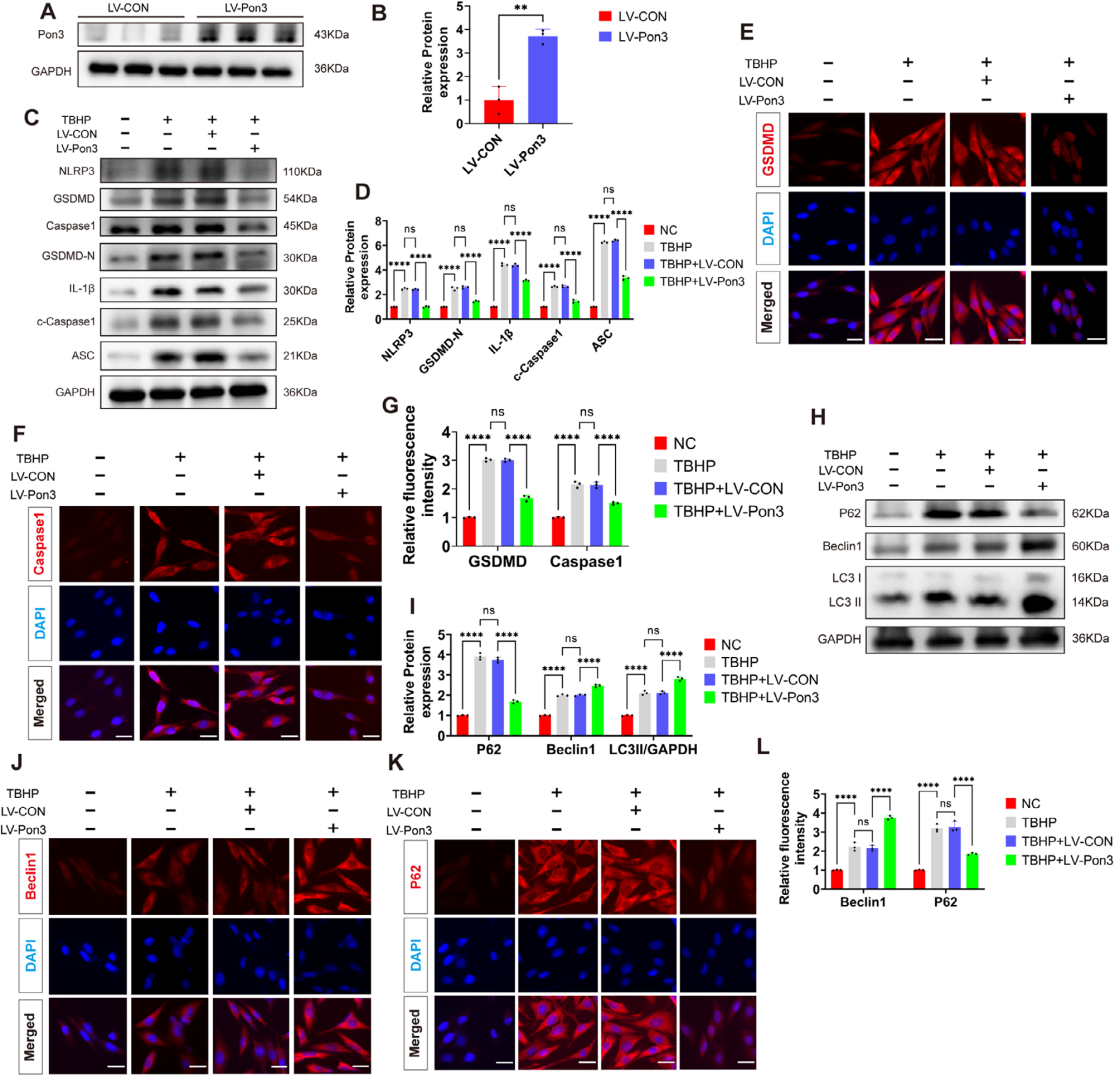
Overexpression of Pon3 Attenuates TBHP‐Induced Pyroptosis and Promotes Autophagy in PC12 Cells. A,B) Western blot detection and quantitative analysis of Pon3 overexpression efficiency in PC12 cells (n = 3). C,D) Western blot detection and quantitative analysis of pyroptosis‐related marker proteins in PC12 cells after TBHP stimulation (n = 3). E–G) Immunofluorescence staining for GSDMD (red), Caspase1 (red), and DAPI (blue), followed by quantitative analysis of relative fluorescence intensity in PC12 cells after TBHP stimulation (n = 3; scale bar: 20 µm). H,I) Western blot detection and quantitative analysis of autophagy‐related marker proteins in PC12 cells after TBHP stimulation (n = 3). J–L) Immunofluorescence staining for Beclin1 (red), P62 (red), and DAPI (blue), followed by quantitative analysis of relative fluorescence intensity in PC12 cells after TBHP stimulation (n = 3; scale bar: 20 µm). Data are presented as mean ± SEM. Significance was determined by two‐tailed unpaired t‐tests or two‐way ANOVA, followed by Tukey's multiple comparisons test. ns: p > 0.05; ** p < 0.01; ****p <0.0001.

Based on our previous findings demonstrating predominant neuronal expression of Pon3 in rats, we established a Pon3‐overexpressing PC12 cell model (**Figure** **4**A,B). According to prior research,^[^ ^33^, ^34^ ^]^ we used Tert – butyl hydroperoxide (TBHP) to establish a pyroptosis model in PC12 cells. Western blot analysis revealed dose‐dependent upregulation of NLRP3, GSDMD, IL‐1β, ASC, P62, and Pon3 (Figure S4A,B, Supporting Information). Moreover, immunofluorescence confirmed the upregulation of Pon3 induced by TBHP (Figure S4D, Supporting Information).

Subsequent live/dead cell staining demonstrated that a TBHP concentration of 400 µm significantly impaired cell viability, leading to cell death (Figure S4G,H, Supporting Information). To verify the reliability of the PC12 cell model, we also compared the expression levels of pyroptosis markers and the neuronal marker β‐III tubulin (TUBB3) in primary neuron models after TBHP treatment. The results showed that in primary neuronal cells, after induction with TBHP, the levels of pyroptosis markers gradually increased with the concentration of TBHP, showing a consistent trend with that in PC12 cells. Additionally, we detected the levels of TUBB3 in the two cell models and found that there was no significant change in TUBB3 levels after TBHP induction in both cell models, indicating that their neuronal characteristics could stably exist after TBHP induction (Figure S5A,B, Supporting Information). Consequently, a TBHP concentration of 200 µm was selected for subsequent cell‐based experiments.

Results from both Western blot and immunofluorescence analyses demonstrated that Pon3 overexpression significantly suppressed the TBHP‐induced upregulation of NLRP3, GSDMD, Caspase‐1, IL‐1β, and ASC levels, thereby effectively attenuating pyroptosis (Figure 4C–G). Furthermore, Western blot and immunofluorescence data revealed that overexpression of Pon3 could not only reduce the elevation of P62 levels induced by TBHP but also further augment the levels of Beclin1 and LC3II following TBHP stimulation (Figure 4H–L). These results strongly indicate that overexpression of Pon3 is capable of promoting autophagy in PC12 cells stimulated by TBHP.

### 2.5 Pon3‐Mediated Autophagy Enhancement Attenuates Pyroptosis and Promotes Functional Recovery in SCI Rats

**Figure 5:**
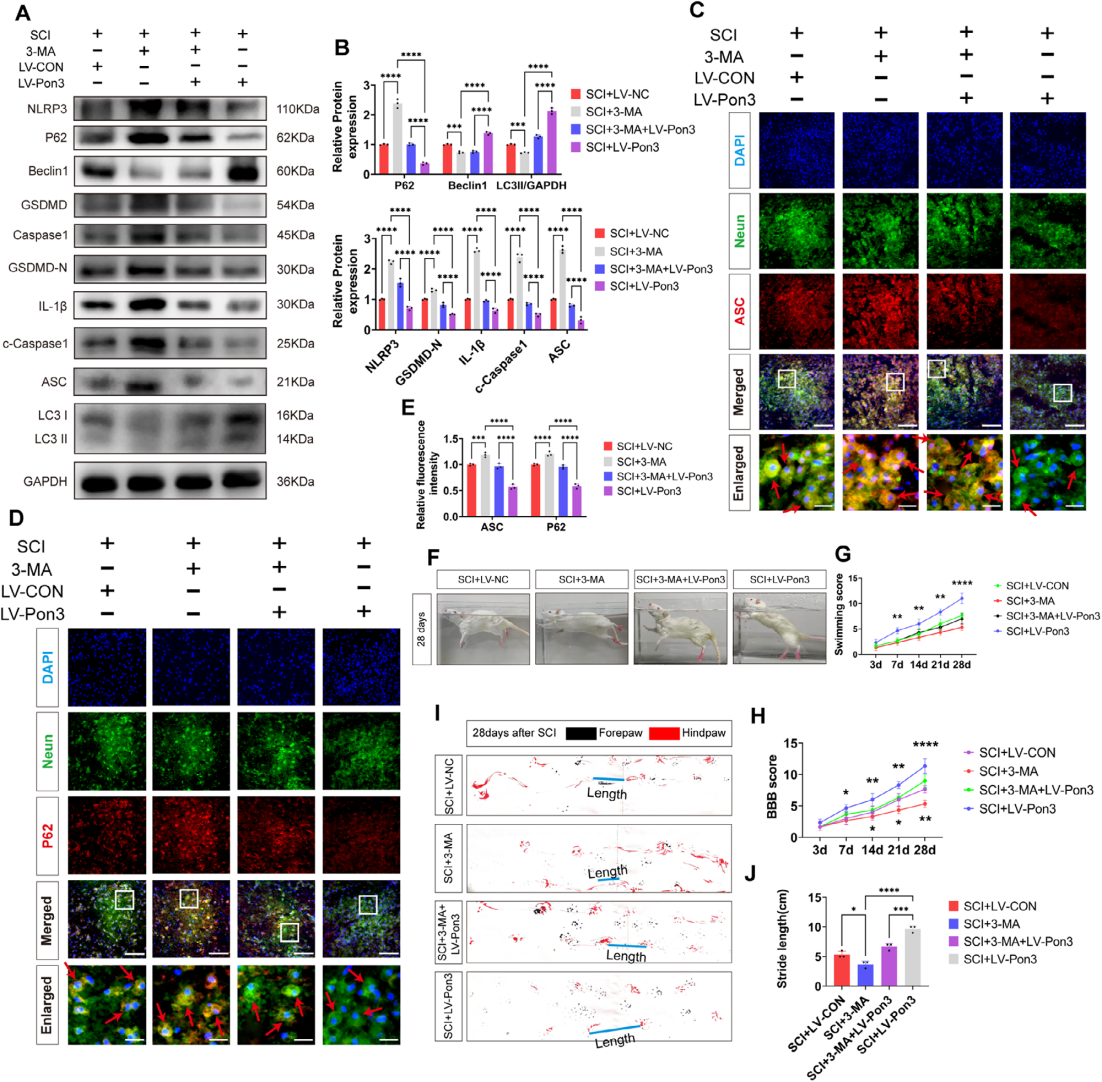
Pon3‐Mediated Autophagy Enhancement Attenuates Pyroptosis and Promotes Functional Recovery in SCI Rats. A,B) Western blot detection and quantitative analysis of pyroptosis and autophagy marker protein expression levels in the spinal cord at 3 days after SCI (n = 3). C–E) Double immunofluorescence staining for ASC (red), P62 (red), and Neun (green) at day 3 after SCI, followed by quantitative analysis of relative fluorescence intensity (n = 3; scale bar: 100 µm in main panels; 20 µm in enlarged insets). F) Representative images of the swimming test at day 28 after SCI. G) Quantitative analysis of swimming test results in rats at different time points after SCI (n = 3). H) Quantitative analysis of BBB scores in rats at different time points after SCI (n = 3). I) Representative footprint images of rats at day 28 after SCI. J) Quantitative analysis of footprint test results in rats at different time points after SCI (n = 3). Data are presented as mean ± SEM. Significance was determined by one‐way or two‐way ANOVA, followed by Tukey's multiple comparisons test. * p < 0.05; ** p < 0.01; *** p <0.001; ****p <0.0001.

To explore the relationship among Pon3, autophagy, and the recovery of motor function in rats, we employed the autophagy inhibitor 3‐methyladenine (3‐MA). First, treatment with 3‐MA further elevated the levels of NLRP3, GSDMD, Caspase1, IL‐1β, ASC, and P62 after SCI, while suppressing the levels of Beclin1 and LC3II. In contrast, overexpression of Pon3 reversed these effects (**Figure** **5**A,B). Immunofluorescence results after co‐localization with neuronal cells also corroborated these findings (Figure 5C–E).

In the behavioral experiments, the results of the swimming test and the BBB locomotor rating scale showed that, starting from the 7th day after SCI, overexpression of Pon3 mitigated the decline in the motor coordination ability and lower limb function of rats induced by the intervention of 3‐MA (Figure 5F–H). Additionally, the footprint test results indicated that, on the 28th day after SCI, overexpression of Pon3 also improved the reduction in the stride length of rats caused by the treatment of 3‐MA (Figure 5I,J). These results suggest that Pon3 can alleviate pyroptosis by promoting autophagy, thereby facilitating the recovery of motor function in rats with SCI.

### 2.6 Pon3 Enhances the Survival of PC12 Cells by Inhibiting TBHP‐Induced Pyroptosis through Promoting Autophagy

**Figure 6:**
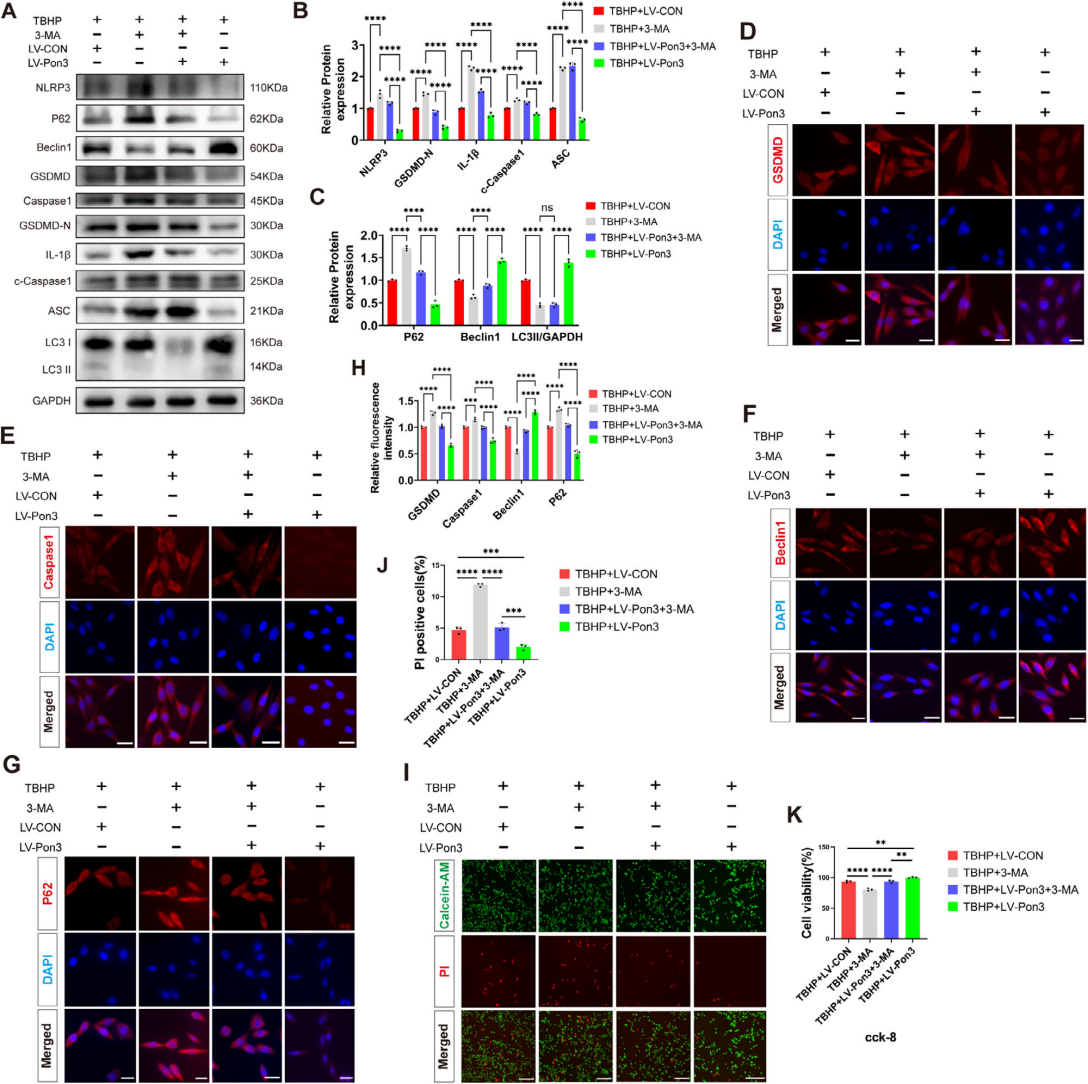
Pon3 Enhances the Survival of PC12 Cells by Inhibiting TBHP‐Induced Pyroptosis through Promoting Autophagy. A–C) Western blot detection and quantitative analysis of pyroptosis and autophagy marker protein expression in PC12 cells co‐stimulated with TBHP and 3‐MA (n = 3). D–H) Immunofluorescence staining for GSDMD (red), Caspase1 (red), Beclin1 (red), P62 (red), and DAPI (blue), followed by quantitative analysis of relative fluorescence intensity in PC12 cells co‐stimulated with TBHP and 3‐MA (n = 3; scale bar: 20 µm). I,J) Live/dead cell staining showing cell survival status and quantitative analysis of dead cells after co‐treatment (n = 3; scale bar: 200 µm). K) Cell viability measured by CCK‐8 assay following TBHP and 3‐MA co‐treatment (n = 3). Data are presented as mean ± SEM. Significance was determined by one‐way or two‐way ANOVA, followed by Tukey's multiple comparisons test. ns: p > 0.05; ** p < 0.01; *** p <0.001; ****p <0.0001.

To investigate whether Pon3 exerts a neuroprotective effect through autophagy in vitro, we applied the autophagy inhibitor 3‐MA to PC12 cells overexpressing Pon3. Western blot analysis revealed that compared to the TBHP+LV‐CON group, the TBHP+3‐MA group showed significant increases in NLRP3, GSDMD, Caspase1, IL‐1β, ASC, and P62, along with reductions in Beclin1 and LC3II. Notably, these changes were reversed in the TBHP+LV‐Pon3 group (**Figure** **6**A–C). The immunofluorescence results further demonstrated that, in the TBHP+3‐MA group, the fluorescence intensities of GSDMD, Caspase1, and P62 were significantly increased, whereas the fluorescence intensity of Beclin1 was significantly decreased, in comparison with the TBHP+LV‐CON group. These changes were also counteracted by Pon3 overexpression (Figure 6D–H). Subsequent live/dead cell staining and CCK‐8 assays demonstrated that compared to the TBHP+LV‐CON group, the TBHP+3‐MA group exhibited significantly increased cell death and reduced viability. These detrimental effects were effectively reversed in the TBHP+LV‐Pon3 group (Figure 6I–K). These results indicate that Pon3 enhances the survival of PC12 cells by promoting autophagy and subsequently reducing pyroptosis.

### 2.7 Identification of S1PR1 as a Downstream Target of Pon3 through mRNA Sequencing

**Figure 7:**
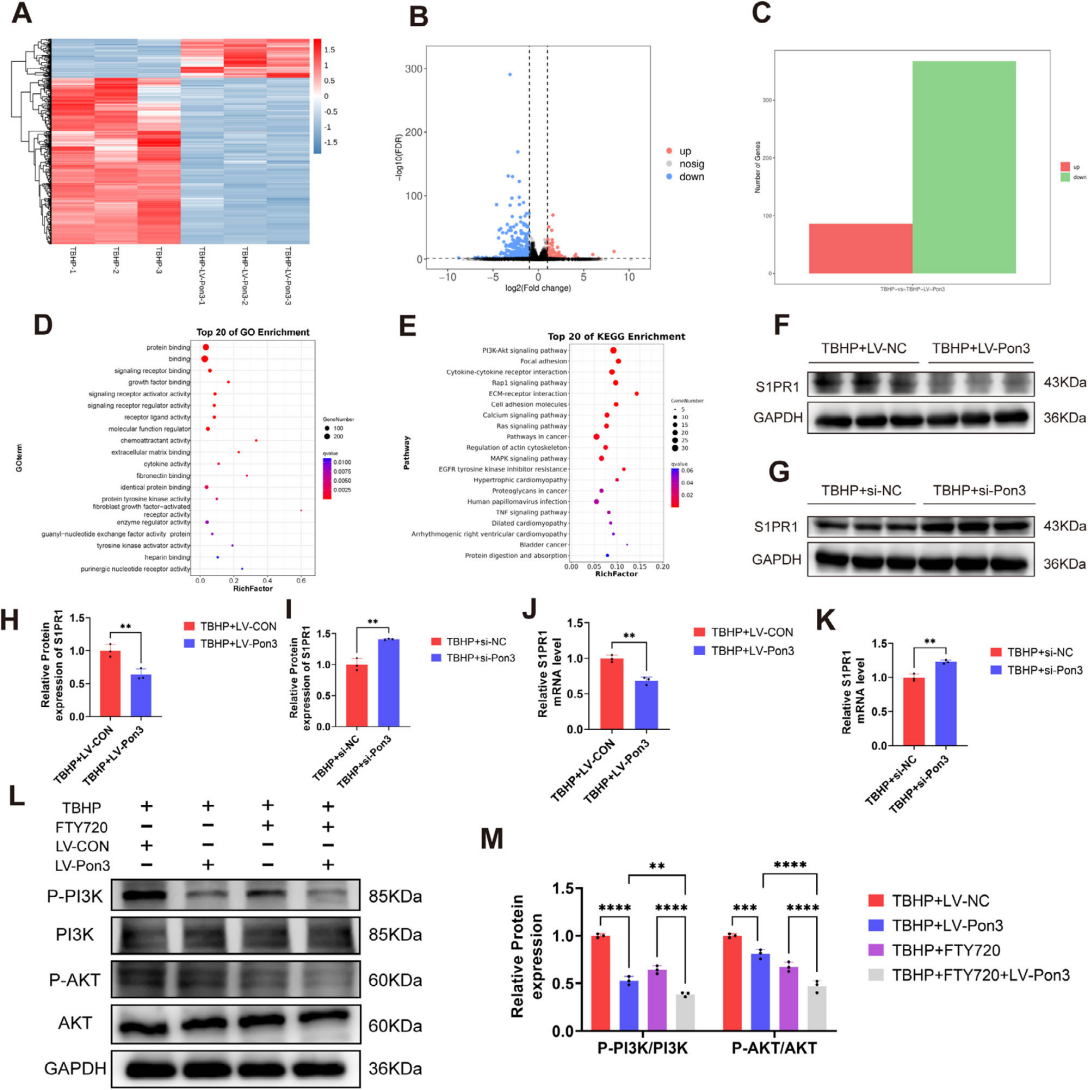
Identification of S1PR1 as a Downstream Target of Pon3 through mRNA Sequencing. A) Heatmap of differentially expressed genes (DEGs) between LV‐NC and LV‐Pon3 groups after TBHP stimulation. B,C) Volcano plot and statistical analysis of DEGs between LV‐NC and LV‐Pon3 treated with TBHP. D,E) GO and KEGG pathway enrichment analysis of DEGs. F–I) Western blot analysis of S1PR1 protein expression following Pon3 overexpression or knockdown, with quantitative analysis (n = 3). J,K) qRT‐PCR detection of S1PR1 expression levels in PC12 Cells (n = 3). L,M) Western blot detection and quantitative analysis of PI3K and AKT protein expression in PC12 cells (n = 3). Data are presented as mean ± SEM. Significance was determined by two‐tailed unpaired t‐tests or one‐way ANOVA, followed by Tukey's multiple comparisons test. ** p < 0.01; *** p <0.001; ****p <0.0001.

To further elucidate the mechanism by which Pon3 alleviates pyroptosis and thereby improves neuronal survival, we identified the downstream target genes of Pon3 through mRNA sequencing. Compared with the TBHP group, heatmaps and volcano plots revealed that in the Pon3 overexpression group, 368 genes were downregulated and 86 genes were upregulated (**Figure** **7**A–C). Gene Ontology (GO) analysis results indicated that the molecular functions of these differentially expressed genes were mainly enriched in aspects such as protein–protein interaction and signal receptor binding (Figure 7D). Kyoto Encyclopedia of Genes and Genomes (KEGG) analysis showed that the functions of these differentially expressed genes were primarily enriched in signaling pathways such as PI3K – Akt, Ras, and MAPK (Figure 7E). Previous studies have demonstrated that signaling pathways like PI3K – Akt and MAPK play crucial roles in pyroptosis.^[^ ^35^, ^36^ ^]^ Therefore, through the results of differential expression analysis, we screened out the S1PR1 gene, which is associated with the PI3K – Akt signaling pathway and the cell cycle. Meanwhile, Western blot and qPCR results confirmed that knockout of Pon3 could increase the level of S1PR1, while overexpression of Pon3 could inhibit the level of S1PR1 (Figure 7F–K). Moreover, Western blot results showed that the protein levels of p‐PI3K and p‐AKT were significantly decreased after Pon3 overexpression. Furthermore, treating Pon3‐overexpressing PC12 cells with FTY720 further reduced the levels of p‐PI3K and p‐AKT (Figure 7L–M). These findings suggest that S1PR1, as a downstream target of Pon3, may play a significant role in pyroptosis.

### 2.8 Pon3 Suppresses S1PR1‐Mediated Pyroptosis and Promotes Autophagy

**Figure 8:**
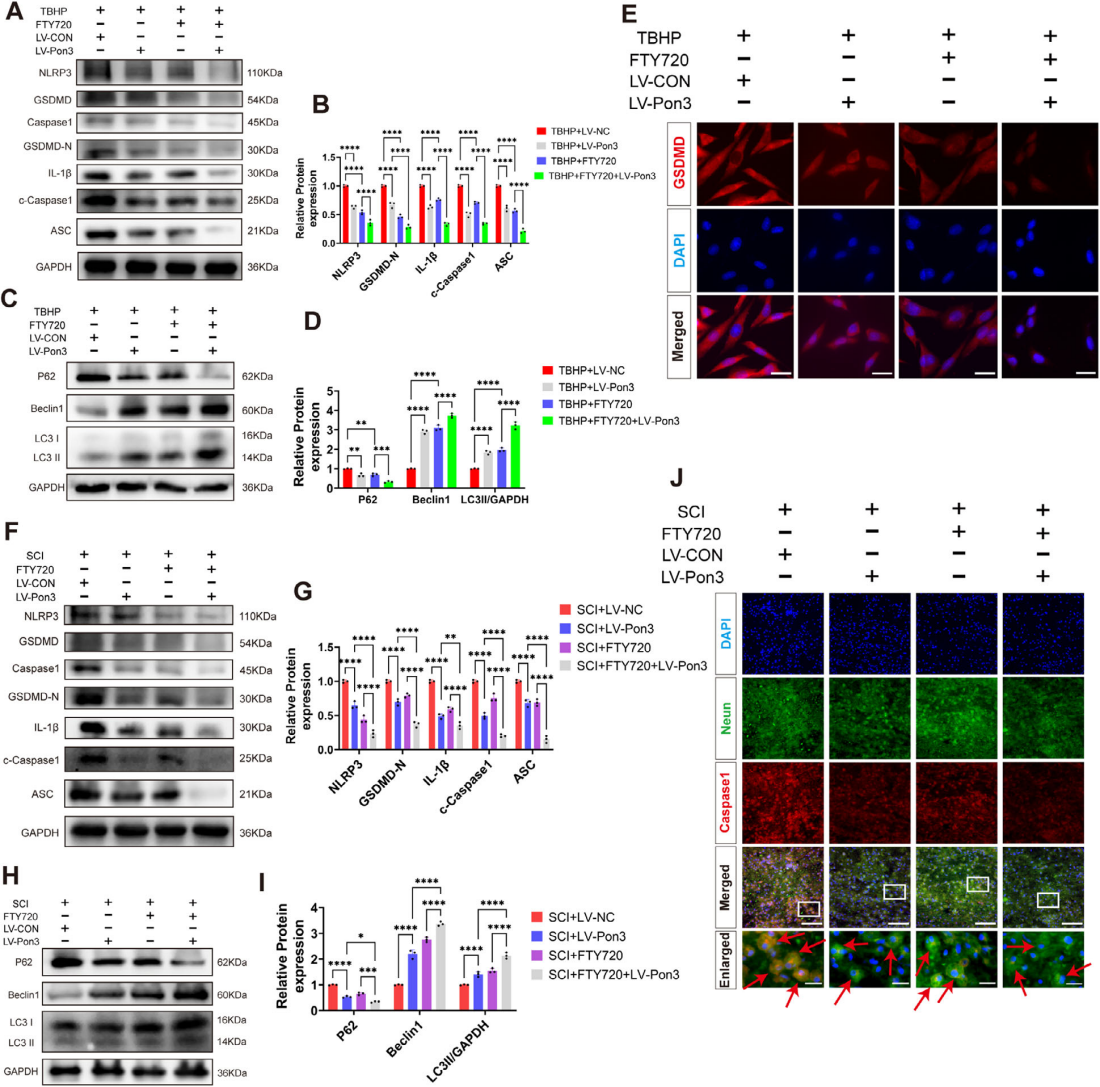
Pon3 Suppresses S1PR1‐Mediated Pyroptosis and Promotes Autophagy. A–D) Western blot detection and quantitative analysis of pyroptosis and autophagy marker protein expression levels in PC12 cells co‐treated with TBHP and FTY720 (n = 3). E) Immunofluorescence detection of the expression levels of GSDMD (red) (scale bar: 20 µm). F–I) Western blot detection and quantitative analysis of pyroptosis and autophagy marker protein expression levels in the spinal cord at day 3 after SCI (n = 3). J) Double immunofluorescence staining of Neun (green) with Caspase1 (red) at 3 days after SCI (scale bar: 100 µm in main panels; 20 µm in enlarged insets). Data are presented as mean ± SEM. Significance was determined by two‐way ANOVA, followed by Tukey's multiple comparisons test. * p < 0.05; ** p < 0.01; *** p <0.001; ****p <0.0001.

To further validate the role of Pon3 exerts its function via S1PR1, we employed Fingolimod (FTY720), an inhibitor of S1PR1. In an in vitro setting, we co – treated PC12 cells overexpressing Pon3 with FTY720(Figure S6A,B, Supporting Information). Western blot analysis and immunofluorescence results revealed that the levels of NLRP3, GSDMD, Caspase1, IL‐1β, ASC, and P62 in the FTY720+TBHP group were significantly lower compared to those in the TBHP+LV‐NC group, while the expressions of Beclin1, LC3II were significantly increased. Notably, the FTY720+TBHP+LV‐Pon3 group further amplified these changes (**Figure** **8**A–E). In an in vivo experiment, we intraperitoneally injected FTY720 to rats that had undergone in – situ spinal cord injection of the virus for Pon3 overexpression. Western blot and immunofluorescence results showed that the levels of NLRP3, GSDMD, Caspase1, IL‐1β, ASC, and P62 in the FTY720+SCI group were significantly reduced compared to those in the SCI + LV‐NC group, and the expressions of Beclin1, LC3II were significantly increased. Similarly, the FTY720+SCI+LV‐Pon3 group further amplified these alterations (Figure 8F–J). These findings indicate that Pon3 can alleviate pyroptosis and promote autophagy by inhibiting S1PR1.

### 2.9 Dnmt1 Promotes Functional Recovery after Spinal Cord Injury by Regulating Pon3 Expression to Attenuate Pyroptosis

**Figure 9:**
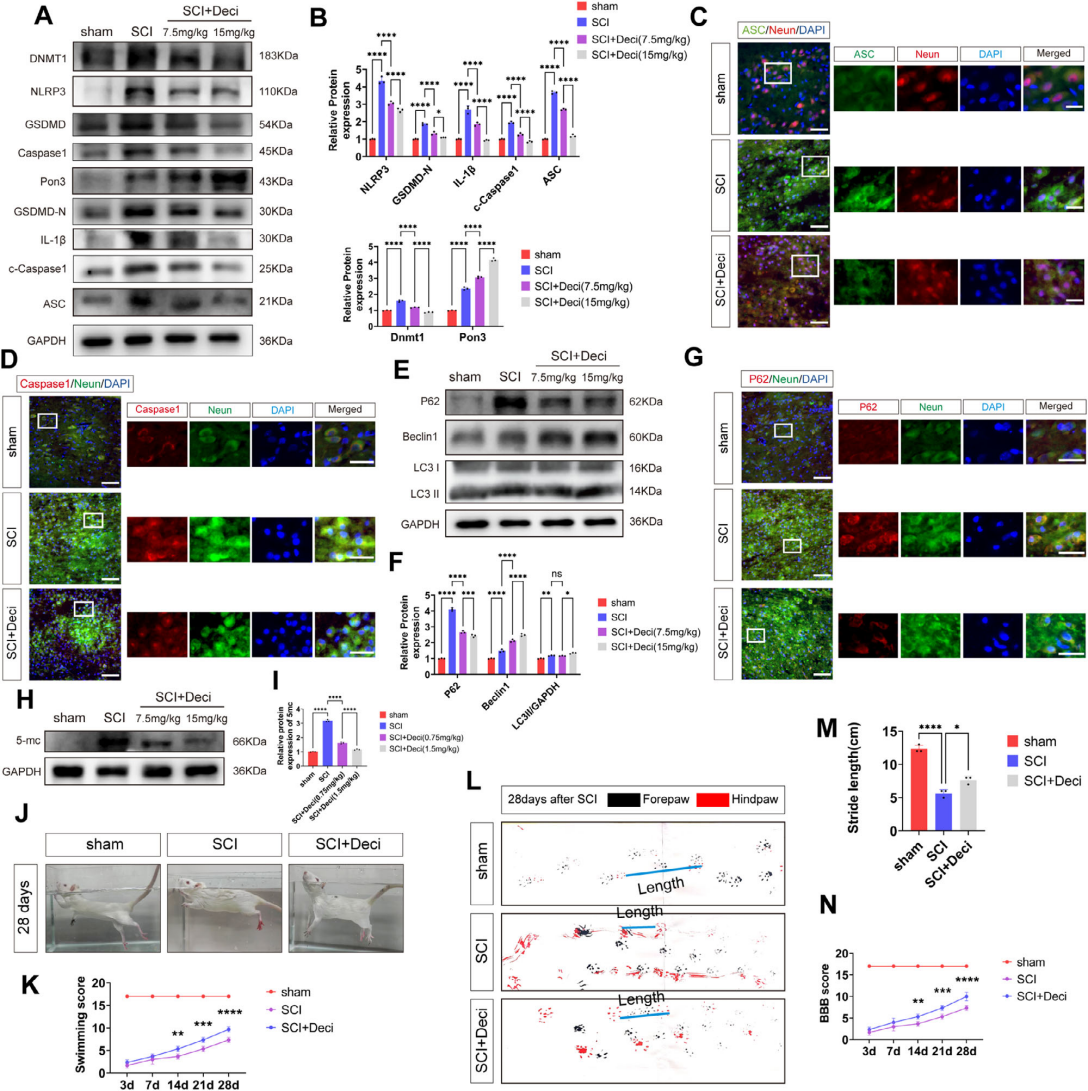
Dnmt1 promotes functional recovery after spinal cord injury by regulating Pon3 expression to attenuate pyroptosis. A,B) Western blot detection and quantitative analysis of Dnmt1, Pon3, and pyroptosis marker protein expression levels in the spinal cord at day 3 after SCI (n = 3). C) Double immunofluorescence staining for ASC (green) and Neun (red) in the spinal cord at day 3 after SCI (scale bar: 50 µm in main panels; 20 µm in enlarged insets). D) Double immunofluorescence staining for Caspase1 (red) and Neun (green) in the spinal cord at day 3 after SCI (scale bar: 50 µm in main panels; 20 µm in enlarged insets). E,F) Western blot detection and quantitative analysis of autophagy marker protein expression levels in the spinal cord at day 3 after SCI (n = 3). G) Double immunofluorescence staining for P62 (red) and NeuN (green) in the spinal cord at day 3 after SCI (scale bar: 50 µm in main panels; 20 µm in enlarged insets). H,I) Western blot detection and quantitative analysis of 5‐mc (methylation product) protein expression levels in the spinal cord at day 3 after SCI (n = 3). J) Representative images of the swimming test at day 28 after SCI. K) Quantitative analysis of swimming test results in rats at different time points after SCI (n = 3). L) Representative footprint images of rats at day 28 after SCI. M) Quantitative analysis of footprint test results in rats at different time points after SCI (n = 3). N) Quantitative analysis of BBB scores in rats at different time points after SCI (n = 3). Data are presented as mean ± SEM. Significance was determined by one‐way or two‐way ANOVA, followed by Tukey's multiple comparisons test. ns: p > 0.05; * p < 0.05; ** p < 0.01; *** p <0.001; ****p <0.0001.

Based on the previous research findings, we observed that after SCI, the levels of Pon3 gradually decreased over time, while the levels of Dnmt1 gradually increased (Figure S7A,B, Supporting Information). Moreover, numerous studies have indicated that the methylation level of Pon3 plays a crucial role in diseases such as cerebral infarction and neurodegeneration.^[^ ^37^, ^38^ ^]^ Therefore, to verify the relationship between Dnmt1 and Pon3, we utilized a Dnmt1 inhibitor (Deci) to explore their interaction. The results of Western blot analysis showed that as the concentration of Deci increased, the level of Dnmt1 gradually decreased, whereas the level of Pon3 gradually increased. Meanwhile, the levels of pyroptosis markers, including NLRP3, GSDMD, Caspase1, IL‐1β, and ASC, also gradually decreased (**Figure** **9**A,B). Immunofluorescence results demonstrated that the expressions of ASC and Caspase1 on neuronal cells significantly increased after SCI, but this effect was reversed after the addition of Deci (Figure 9C,D). In addition, Western blot results indicated that the addition of Deci reduced the level of the autophagic substrate protein P62 after SCI. Conversely, it further increased the levels of autophagy‐related markers Beclin1 and LC3II (Figure 9E,F). The immunofluorescence results also showed that the expression of P62 in neuronal cells significantly increased after SCI, and this increase was reversed after the addition of Deci (Figure 9G). According to previous research,^[^ ^39^ ^]^ to verify the methylation level of rats after the addition of Deci, we detected the methylation product 5‐mc by Western blot. The results showed that the addition of Deci significantly reduced the level of 5‐mc after SCI (Figure 9H,I). These results demonstrate that Dnmt1 regulates Pon3 expression through modulation of DNA methylation, thereby attenuating pyroptosis following SCI.

Furthermore, the results of the swimming test, gait analysis, and the BBB locomotor rating scale indicated that, compared with the SCI group, the SCI + Deci group significantly improved the lower limb motor coordination, stride length, and functional recovery of rats with SCI (Figure 9J–N). Meanwhile, HE staining and Nissl staining showed that the intervention of Deci significantly reduced the injury area and increased the number of surviving neurons (Figure S8A–E, Supporting Information). These results suggest that Dnmt1 can improve the functional recovery after SCI by regulating the expression of Pon3 and alleviating pyroptosis. However, whether Dnmt1 exerts its function solely through the regulation of Pon3 requires further verification by in vitro experiments.

### 2.10 Dnmt1 Enhances PC12 Cell Survival by Suppressing Pyroptosis via Pon3 Regulation

**Figure 10:**
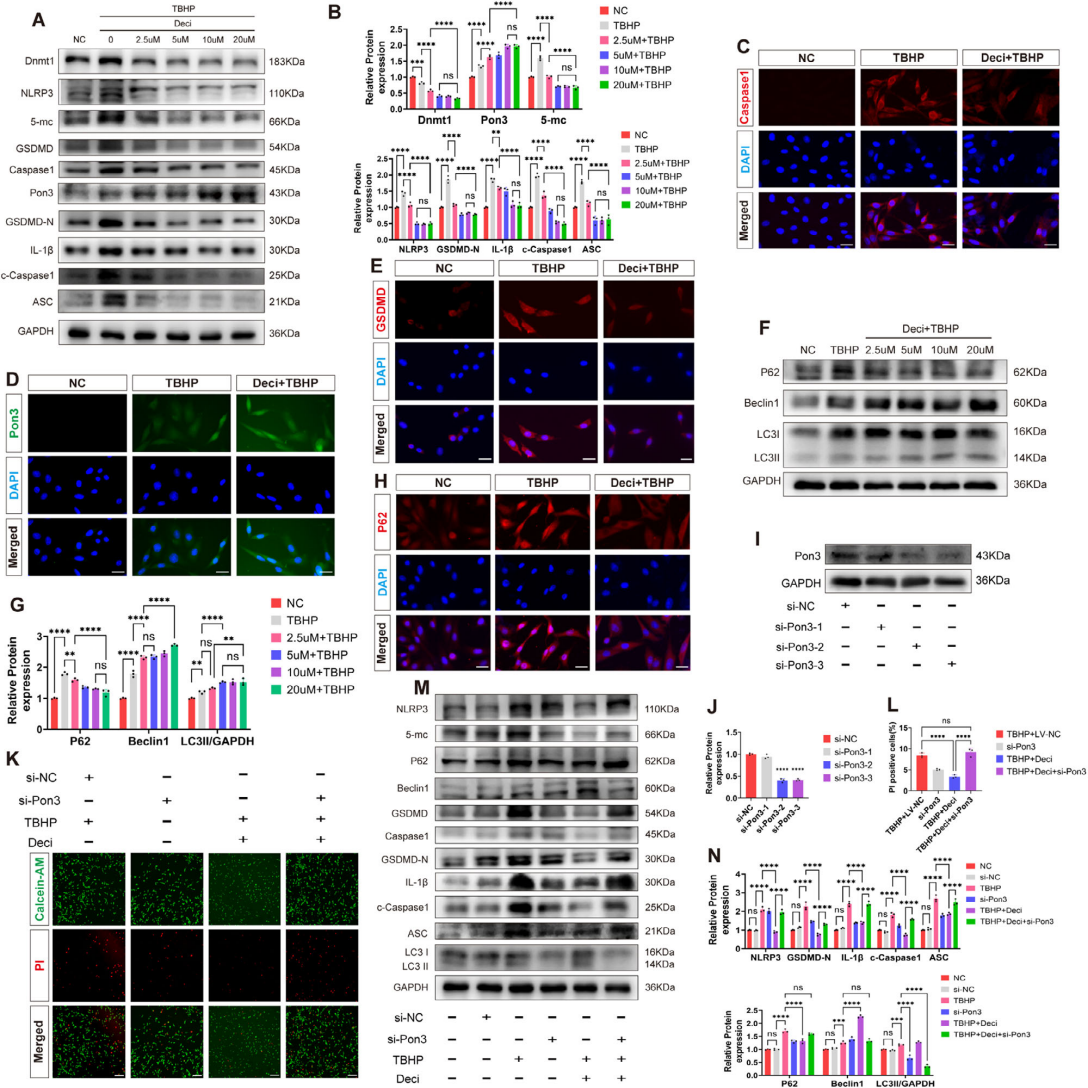
Dnmt1 Enhances PC12 Cell Survival by Suppressing Pyroptosis via Pon3 Regulation. A,B) Western blot detection and quantitative analysis of protein expression levels for Dnmt1, 5‐mc, Pon3, and pyroptosis markers in PC12 cells co‐treated with TBHP and Deci (n = 3). C–E) Immunofluorescence staining of PC12 cells after co‐treatment with TBHP and Deci, using Caspase1 (red), Pon3 (green), GSDMD (red), and DAPI (blue) (scale bar: 20 µm). F,G) Western blot analysis and quantitative evaluation of autophagy marker protein expression in PC12 cells co‐treated with TBHP and Deci (n = 3). H) Immunofluorescence staining of P62 (red) and DAPI (blue) in PC12 cells co‐treated with TBHP and Deci (scale bar: 20 µm). I,J) Western blot analysis and quantification of Pon3 knockdown efficiency via si‐RNA transfection in PC12 cells (n = 3). K,L) Live/dead cell staining to assess cell survival status and quantify dead cells in PC12 cells co‐stimulated with TBHP and Deci after transfection (n = 3; scale bar: 200 µm). M,N) Western blot analysis and quantitative assessment of 5‐mc, pyroptosis markers, and autophagy marker protein expression in PC12 cells co‐stimulated with TBHP and Deci after transfection (n = 3). Data are presented as mean ± SEM. Significance was determined by one‐way or two‐way ANOVA, followed by Tukey's multiple comparisons test. ns: p > 0.05; ** p < 0.01; *** p <0.001; ****p <0.0001.

To further elucidate the neuroprotective mechanism of Dnmt1 in PC12 cells, we assessed the expression levels of key molecules via Western blot and immunofluorescence. The results demonstrated a dose‐dependent decrease in NLRP3, GSDMD, Caspase1, IL‐1β, ASC, P62, and 5‐mC levels with increasing Deci concentrations, while Beclin1 and LC3II expression progressively increased (**Figure** **10**A–H).

To investigate the relationship between Dnmt1 and autophagy, we applied the autophagy inhibitor 3‐MA in vitro. To further verify that 3‐MA effectively inhibits autophagy in PC12 cells, we transfected PC12 cells with stubRFP‐sensGFP‐LC3 lentivirus. The results showed that compared with the TBHP group, Dnmt1 inhibition significantly promoted autophagic flux, while the addition of 3‐MA reversed this promoting effect and inhibited autophagic flux (Figure S9A, Supporting Information). Western blot and immunofluorescence results indicated that 3‐MA abolished the inhibitory effects of Deci on NLRP3, GSDMD, Caspase1, IL‐1β, ASC, and P62, as well as the upregulatory effect on LC3II. This suggests that Dnmt1 alleviates pyroptosis by enhancing the autophagic flux (Figure S10A–G, Supporting Information).

To investigate the level of Pon3 promoter methylation regulated by Dnmt1, we detected the methylation level of Pon3 promoter after Dnmt1 inhibition by Methylation‐Specific PCR (MSP). The results indicated that compared with the TBHP group, Dnmt1 inhibition significantly reduced the methylation level of Pon3 and increased the unmethylated level (Figure S11A, Supporting Information).

Subsequently, to demonstrate that Dnmt1 exerts its neuroprotective effect solely through the regulation of Pon3, we knocked down Pon3 using small interfering RNA (Figure 10I,J). The live/dead cell staining results showed that the number of dead cells in the TBHP+Deci group was significantly lower than that in the TBHP group. In contrast, the TBHP+Deci+si‐Pon3 group exhibited an increased number of dead cells (Figure 10K,L). Meanwhile, Western blot results revealed that the TBHP+Deci+si‐Pon3 group reversed the changes in protein levels that were induced by the TBHP+Deci group (Figure 10M,N). These findings collectively demonstrate that Dnmt1‐mediated neuroprotection depends predominantly on Pon3 regulation.

## 3 Discussion

SCI can be divided into mechanical injury and secondary injury, which is mainly characterized by various pathological reactions subsequently.^[^ ^40^ ^]^ In particular, during the pathological reactions, neuroinflammation and cell death play crucial roles.^[^ ^41^, ^42^ ^]^ Pyroptosis is a caspase‐dependent inflammatory cell death pathway (mediated by Caspase‐1/4/5/11) characterized by the proteolytic maturation and secretion of pro‐inflammatory cytokines, including IL‐1β and IL‐18, culminating in lytic cellular demise. Autophagy is a process of degrading damaged organelles and related proteins through lysosomes, and recycling the decomposed components. Under stress conditions, autophagy can be significantly upregulated to alleviate stress‐induced damage and help cells survive.^[^ ^43^ ^]^ Earlier investigations have demonstrated that the PI3K/AKT signaling pathway is involved in the pyroptosis of neurons following SCI, while autophagy can exert a protective effect against it.^[^ ^44^ ^]^ Additionally, research has shown that Advanced Oxidation Protein Products mitigate post‐SCI cellular pyroptosis by modulating the MAPK cascade.^[^ ^45^ ^]^ Given the demonstrated involvement of pyroptosis in SCI pathogenesis, identifying key regulatory nodes in this process emerges as a crucial research direction.

Characterized by their antioxidant, anti‐inflammatory properties, and the ability to inhibit cell proliferation, PONs demonstrate therapeutic potential in CNS disorders, heart disease, and cancer.^[^ ^46^ ^]^ Among them, Pon3 is predominantly hepatically‐derived, undergoing systemic distribution via apolipoprotein/HDL complexes to target tissues. Furthermore,^[^ ^47^ ^]^ studies have demonstrated that Pon3 is expressed in both the gray and white matter regions of the spinal cord, as well as in the brain, which provides a basis for the improvement of related neurological disorders.^[^ ^20^ ^]^ Meanwhile, other studies have demonstrated that Pon3 inhibits cell death caused by inflammation by suppressing the production of superoxide.^[^ ^21^ ^]^ In this study, we screened out the Pon3 gene through bioinformatics analysis. Subsequently, our experiments demonstrated that Pon3 can improve the prognosis of SCI alleviating pyroptosis. These results indicate that targeting Pon3 may represent a viable therapeutic strategy for SCI intervention.

^[^ ^20^ ^]^Studies have shown that Pon3 can exert antioxidant effects through microglia, thereby playing a role in Alzheimer's disease and other neurodegenerative diseases. In this study, Pon3, the screened target, was first investigated for its relationship with the prognosis of SCI. We found that after SCI, the level of Pon3 increased to its peak on the first day, and then gradually decreased over time. This change may be attributed to the fact that the early upregulation of Pon3 represents an endogenous protective response, where secreted endogenous Pon3 participates in the early stress response. The subsequent sustained decline of Pon3 indicates that epigenetic silencing becomes dominant. After spinal cord injury, the methylation level of Pon3 gradually increases, leading to the peak of Pon3 expression on day 1 followed by a continuous decline. Additionally, we found that the change in Pon3 is most pronounced in neurons following SCI. Subsequently, we discovered that overexpression of Pon3 could reduce the lesion area in rats with SCI and decrease the loss of neurons, thereby improving the lower limb motor coordination and function of the rats. This finding demonstrates that Pon3 has the potential to be a promising target for the treatment of SCI. Subsequently, using both in vitro and in vivo models, we quantified key pyroptosis markers (NLRP3, GSDMD, Caspase1, IL‐1β, ASC), the autophagic substrate P62, and autophagy‐related proteins (Beclin1, LC3II) to investigate Pon3's regulatory role in pyroptosis and its mechanistic basis. We found that upon overexpression of Pon3, the concentrations of markers associated with pyroptosis and the autophagic substrate were significantly reduced, while the levels of autophagy‐related markers were further increased. These results suggest that overexpression of Pon3 can alleviate pyroptosis induced by SCI and promote autophagy. To verify the relationship among Pon3, autophagy, and SCI, we used the autophagy inhibitor 3‐MA and found that 3‐MA reversed the changes induced by the overexpression of Pon3. This result indicates that Pon3 alleviates pyroptosis by promoting autophagy, which further improves the prognosis of rats with SCI and the survival of PC12 cells. Subsequently, to elucidate the mechanism between Pon3 and pyroptosis, transcriptomic sequencing revealed that S1PR1 might be a downstream target of Pon3. Therefore, we used the S1PR1 inhibitor FTY720 for subsequent experiments.^[^ ^48^ ^]^ Previous studies have shown that FTY720 can cross the blood‐brain barrier to exert neuroprotective effects. Subsequently, we confirmed through in vivo and in vitro experiments that Pon3 improves the prognosis of SCI by inhibiting S1PR1‐mediated pyroptosis, thus providing a novel perspective for the pathological treatment of SCI.

As the primary executor of methylation maintenance, Dnmt1 governs epigenetic regulation essential for normal development, genome protection, and disease etiology in mammalian systems. Recent studies reveal its oncogenic role via epigenetic silencing of tumor suppressors, thereby promoting neoplastic transformation, metastatic potential, and therapeutic recalcitrance.^[^ ^49^ ^]^ In neurological contexts, Dnmt1 overexpression exacerbates neuronal apoptosis, while its inhibition protects motor neurons in amyotrophic lateral sclerosis (ALS) models and mitigates cerebral ischemic injury.^[^ ^39^ ^]^ However, the functional significance of Dnmt1 in SCI and its potential regulation of Pon3 expression remain unexplored. In this study, we used the Dnmt1 inhibitor Deci for subsequent experiments.^[^ ^50^ ^]^ Previous studies have shown that Deci can effectively cross the blood‐brain barrier. Our experimental data demonstrate that Dnmt1 suppression reduces pyroptosis markers while concurrently enhancing autophagic flux indicators across both animal and cellular models. Additionally, 3‐MA treatment established that Dnmt1 suppression attenuates pyroptosis via enhanced autophagic flux, contributing to improved SCI outcomes. Subsequently, to verify the relationship between Dnmt1 and Pon3, we knocked out Pon3 in vitro and found that the inhibition of Dnmt1 significantly reduced the level of the methylation product 5‐mc, while the expression of Pon3 increased significantly. This change was reversed after Pon3 was knocked out, indicating that Dnmt1 can regulate the expression of Pon3 by modulating the methylation level of Pon3. This, in turn, promotes autophagy, alleviates pyroptosis, and ultimately improves the prognosis of SCI.

Collectively, our findings provide substantial mechanistic insights with potential clinical translational value for SCI treatment. However, several limitations warrant consideration: First, the potential involvement of Pon3 in other SCI‐related pathological processes remains unexplored, and while lentivirus‐mediated gene editing technology has brought enormous application potential, off‐target effects remain a key issue restricting its development. Although there are currently multiple strategies to reduce off‐target risks, completely eliminating off‐target effects still remains challenging. Second, we should acknowledge that in vitro models cannot fully mimic the complex microenvironment following SCI. Furthermore, while we focused on Dnmt1‐mediated regulation of Pon3, other DNA methyltransferases (such as Dnmt3a/3b) may similarly influence Pon3 expression and merit examination. Finally, in our previous findings, we observed a trend toward increased Pon3 expression in microglia following SCI. Consequently, the role of Pon3 in microglia warrants further investigation, and in the future, we can conduct further experimental investigations to address these aspects comprehensively.

## 4 Conclusion

In conclusion, our research has demonstrated that Pon3 is a pivotal factor in improving the prognosis of SCI. Moreover, Dnmt1 can regulate the expression of Pon3 by modulating the methylation level, thereby inhibiting S1PR1‐mediated pyroptosis and ultimately improving the prognosis of SCI. This result suggests that the regulation of the Dnmt1‐Pon3‐S1PR1 axis has the potential to serve as a viable approach for the treatment of SCI.

## 5 Experimental Section

### Data Acquisition and Differential Gene Expression Analysis

Two datasets (GSE20907 and GSE45006) were retrieved from the GEO database (https://www.ncbi.nlm.nih.gov/geo/). The GSE20907 dataset comprised 12 normal and 12 SCI samples from rat models, while GSE45006 included 4 normal and 20 SCI samples. Differential gene expression analysis was performed using R software (v4.4.1, https://www.r‐project.org/) with the limma package. Genes with an adjusted P‐value<0.05 and |log_2_FC|>1 were considered statistically significant.

### Animals

To assist the urination of rats after SCI and prevent urinary tract infections, adult female Sprague‐Dawley (SD) rats aged 8 to 12 weeks, weighing approximately 200 to 250 grams, were selected for this experiment. These rats were provided with standard water and food and housed in a facility with a light‐dark cycle. All animal procedures were performed in accordance with protocols approved by the Institutional Animal Care and Use Committee of Zhujiang Hospital, Southern Medical University (ethics Number: LAEC‐2024–193).

### Animal Experimental Design and Lentivirus Injection

According to previous studies, sodium pentobarbital (70 mg kg^−1^) was intraperitoneally injected into the rats to induce general anesthesia.^[^ ^51^, ^52^ ^]^ The T9 vertebral lamina was carefully resected to expose the underlying spinal cord segment. The SCI model was generated using a computer‐controlled spinal cord impactor (RWD, China). The impactor tip was precisely positioned over the T9 vertebral level before delivering a standardized contusion (2.5 m/s velocity, 1.4 mm displacement, 1 s dwell time). Once spinal cord congestion and edema were observed, along with the presence of tail‐flick reflex and paralysis of both lower limbs, these observations confirmed successful model induction. The muscles were then sutured layer by layer, and the surgical site was disinfected with povidone‐iodine. The rats were subsequently placed on a pre‐warmed mat. To assist the rats with urination, their bladders were massaged twice a day.

Animals were randomized into ten experimental cohorts (n = 6):the sham operation group, the SCI group, the SCI+LV‐CON group, the SCI+LV‐Pon3 group, the SCI+3‐MA group, the SCI+3‐MA+LV‐Pon3 group, the SCI+FTY720 group, the SCI+FTY720+LV‐Pon3 group, the SCI+Deci (7.5 mg/kg) group, and the SCI+Deci (15 mg kg^−1^) group. Among them, the lentivirus for overexpressing Pon3 (LV‐Pon3) and the negative control lentivirus (LV‐CON) were injected in situ into the spinal cord 7 days prior to the surgery. 3‐MA (KKL MED, KM17736), FTY720(MCE, HY‐12005), and Deci (TargetMol, T1508) were administered intraperitoneally on the day of the surgery, once per day over a continuous span of three days.

The lentiviral vector LV5 (EF‐1a/GFP/Puro) for overexpressing Pon3 was purchased from GenePharma. 7 days prior to SCI, 10 µL of the lentivirus for Pon3 overexpression or the negative control lentivirus was injected in situ into the spinal cord using a microsyringe at a rate of 5 µL min^−1^. To ensure proper viral delivery and prevent leakage, the needle was maintained in situ for 1 min post‐injection, followed by 10 min of gentle compression at the surgical site.

### Behavioral Tests

*Basso‐Beattie‐Bresnahan (BBB) Rating Scale*: According to previous studies,^[^ ^53^ ^]^ neuromotor function of the hindlimbs of rats after SCI was evaluated at different time points after SCI. Briefly, animals were video‐recorded in an open‐field apparatus. Subsequently, three trained researchers scored the rats' motor function. This standardized scale quantifies motor recovery from 0 (complete paralysis) to 21 (normal ambulation).

*Footprint Analysis*: As previously mentioned,^[^ ^54^ ^]^ the hindlimb motor coordination and recovery of rats 28 days after SCI were evaluated. Briefly, the forepaws and hindpaws of the rats were painted with different – colored dyes. Then, the rats were placed on a runway that was 1 m long and 10 cm wide and covered with white paper, and were allowed to walk on the runway. Finally, a quantitative analysis of the footprints was performed.

*Swimming Test*: Previous studies have indicated that the Louisville Swim Score (LSS) is a well‐recognized method for assessing neuromotor functional restoration following SCI.^[^ ^55^ ^]^ The LSS ranges from 0 to 17 points, where 0–5 points indicate poor swimming ability and 12–17 points represent strong swimming ability. Briefly, at different time points after SCI, the rats were placed in a glass tank filled with water and made to swim from one end to the other. Their swimming performance was recorded using professional equipment, and then three trained researchers scored them according to the LSS criteria.

### Tissue Preparation

According to the previous research, rats were anesthetized using the previously described anesthesia method. Subsequently, the diaphragm was incised to expose the heart, and the right atrial appendage was carefully cut open. First, 40 mL of phosphate – buffered saline (PBS) was perfused through the rat heart, followed by 40 mL of 4% paraformaldehyde (PFA). Thereafter, the spinal cord tissue at the injury segment was excised and placed in 4% PFA for fixation at 4 °C in a refrigerator for 1 day. Subsequently, after following sucrose gradient treatment (10/20/30%), samples were snap‐frozen in OCT and sectioned into 12‐µm‐thick slices using a cryostat for subsequent experiments.

### Hematoxylin–Eosin (HE) Staining

Cryosections were equilibrated to room temperature for 30 min, fixed in 4% PFA for 10 min, and rinsed under running water. Hematoxylin and eosin (HE) staining was performed, followed by graded alcohol dehydration, xylene clearing (3 min), and neutral resin mounting. Stained sections were imaged using microscopy.

### Nissl Staining

Cryosections were acclimated to room temperature (10 min), fixed in 4% PFA (10 min), and rinsed with water. After staining with 0.5% toluidine blue and differentiation, sections were oven‐dried (65 °C) and mounted with neutral gum for microscopic observation.

### Cell Experiment Plan

PC12 cells (Shanghai Cell Research Center) were cultured in DMEM containing 10% FBS and 1% antibiotic‐antimycotic solution, and incubated at 37 °C in a humidified 5% CO_2_ atmosphere. As previously described, to induce cell pyroptosis, PC12 cells were stimulated with TBHP (Macklin, B802372‐50ml) at concentrations of 50, 100, and 200 µm for 4 h, respectively. The previous experiments indicated that a concentration of 200 µm could be used for subsequent cell experiments. Cells in the control group were left untreated. The cells were divided into the following groups: the control group, the TBHP group, the TBHP+LV‐CON group, the TBHP+LV‐Pon3 group, the TBHP+3‐MA group, the TBHP+3‐MA+LV‐Pon3 group, the TBHP+FTY720 group, the TBHP+FTY720+LV‐Pon3 group, and the TBHP+Deci group. 3‐MA, FTY720, and Deci were added to the cell culture dishes along with the medium, and subsequent experiments were carried out 1 day later.

### Cell Viability

Cell viability was determined via the CCK‐8 assay (NCM Biotech, C6005). PC12 cells were plated in 96‐well plates at an optimal density and treated for 24 h. After treatment, 10% CCK‐8 reagent in fresh medium was added, followed by a 2 h incubation at 37 °C. Absorbance readings were taken at 450 nm using a microplate reader.

### Primary Neuronal Cultures

To isolate and culture primary spinal neurons, spinal cord tissues were collected from SD rats within 24 h of birth. After careful removal of the meninges and blood vessels, the tissues were digested in 0.25% trypsin for 20 min. Digestion was terminated using DMEM/F12 medium supplemented with 10% FBS, followed by centrifugation (5 min) to pellet the cells. The supernatant was discarded, and the cells were resuspended in Neurobasal medium containing 2% B27, penicillin, and streptomycin. Finally, the cell suspension was plated onto poly‐L‐lysine‐coated culture dishes.

### Cytotoxicity Assay

PC12 cells were seeded in 96‐well plates at a predetermined density. After being treated for 24 h, the cytotoxicity of the cells was detected using the Calcein‐AM/PI double staining kit (Beyotime, C2015S). Briefly, the detection buffer was mixed with the Calcein‐AM/PI working solution. Following an incubation for 30 min at 37 °C in the dark, live cells (displaying green fluorescence due to Calcein‐AM) and dead cells (showing red fluorescence from PI) were observed under a fluorescence microscope.

### Western Blot Analysis

Spinal cord tissues were harvested from rats at 3 days post‐SCI, or PC12 cells were collected after 24 h of treatment. Protein extraction was performed using RIPA lysis buffer containing protease inhibitors, followed by quantification with the BCA assay. Equal amounts (20 µg) of protein were separated on 8% or 12% SDS‐PAGE gels and then transferred to NC or PVDF membranes. The membranes were blocked with 5% BSA for 90 min before further processing. Then, they were incubated overnight at 4°C with rabbit anti‐Dnmt1 (HUABIO, ET1702‐77, 1:1000), rabbit anti‐NLRP3(Proteintech, 30109‐1‐AP, 1:1000), rabbit anti‐SQSTM1/P62(Affinity, AF5384, 1:1000), rabbit anti‐Beclin1 (ABclonal, A21191, 1:1000), rabbit anti‐TUBB3(Zenbio, R23620, 1:1000), rabbit anti‐p‐PI3K(Zenbio, 310164, 1:1000), rabbit anti‐PI3K(Zenbio, R22768, 1:1000), rabbit anti‐p‐AKT(Zenbio, 310021, 1:1000), rabbit anti‐AKT(Zenbio, R23412, 1:1000), rabbit anti‐cleaved‐Caspase1(Zenbio, 341030, 1:1000), mouse anti‐Pon3 (SANTACRUZ, sc‐515603, 1:1000), rabbit anti‐GSDMD (Proteintech, 20770‐1‐AP, 1:1000), rabbit anti‐Caspase1 (Affinity, AF5418, 1:1000), rabbit anti‐S1PR1 (HUABIO, ET1703‐27, 1:1000), rabbit anti‐IL‐1β (bayjoint, IPB0002, 1:1000), rabbit anti‐ASC (Affinity, DF6304, 1:1000), rabbit anti‐LC3B (Novus, NB100‐2220, 1:1000), and mouse anti‐GAPDH (Proteintech, 60004‐1‐Ig, 1:1000), respectively. After washing the membrane three times with TBST, it was incubated with HRP (Goat anti Rabbit) (abbkine, A21020, 1:10000) or HRP (Goat anti mouse) (abbkine, A21010, 1:10000) for 1 h, followed by another three washes with TBST. Finally, the membrane was visualized using an ECL reagent (NCM Biotech, P10100). The optical density of protein bands was measured using ImageJ software.

### Immunofluorescence Staining

Tissue sections were equilibrated at room temperature for 20 min, while treated PC12 cells were fixed in 4% PFA for 10 min. All samples were then permeabilized with 0.3% Triton X‐100 (10 min) and blocked using 5% BSA (45 min). Then, add rabbit anti‐P62 (1:100), rabbit anti‐Beclin1 (1:100), mouse anti‐Pon3 (1:100), rabbit anti‐GSDMD (1:100), rabbit anti‐Caspase1 (1:100), mouse anti‐ASC (1:100) or rabbit anti‐ASC (1:100), mouse anti‐Neun (HUABIO, HA601111, 1:100) or rabbit anti‐Neun (Zenbio, R381075, 1:100), rabbit anti‐GFAP (bayjoint, IPB3211, 1:100), and rabbit anti‐IBA1 (bioworld, BS40716‐25, 1:100), and incubate them overnight at 4°C. After washing the samples three times with PBS containing Tween 20 (PBST), incubate them with FITC(Goat anti mouse) (abbkine, A22110, 1:500) or Cy3 (Goat anti Rabbit) (abbkine, A22220, 1:500) in the dark for 1 h. Wash the samples three times with PBST, and then stain them with a solution containing DAPI for 10 min. After that, mount the samples with an anti‐fluorescence quenching agent. Finally, take images using a confocal microscope, and semi‐quantify the fluorescence intensity with Image J.

### mRNA Sequencing and Data Analysis

After a 24 h treatment, PC12 cell RNA was extracted with Trizol, and then sequenced on the Illumina platform following the manufacturer's instructions. Differentially expressed genes (DEGs) were identified using the DESeq2 package, with significance thresholds set at |log_2_FC|>1 and P‐value < 0.05. Functional enrichment analysis was performed with topGO (v2.36.0) for Gene Ontology (GO), while pathway analysis was conducted using clusterProfiler (v3.12.0) to assess KEGG pathway enrichment of the DEGs.

### Small Interfering RNA Transfection

siRNAs targeting rat Pon3 (si‐Pon3) and a negative control siRNA (si‐NC) were designed and synthesized by Genome Company. The sequences of Pon3‐specific siRNAs were:

Pon3 (r)‐si‐1: 5’‐GGCUCUGAAGAUAUUGAUA‐3’
Pon3 (r)‐si‐2: 5’‐GGACUAAAAUAUCCAGGUA‐3’
Pon3 (r)‐si‐3: 5’‐GAGCAGUUCUAUGCUACAA‐3’

For transfection, Lipofectamine 2000(Invitrogen, 11668‐027) (diluted in Opti‐MEM) and siRNA (diluted in Opti‐MEM) were mixed and incubated for 20 min at room temperature. The transfection complex was introduced to the culture medium. Following a 6‐h incubation period, the medium was refreshed, and cells were maintained for another 48 h prior to further analysis.

### Double‐Labeled Lentivirus stubRFP‐sensGFP‐LC3 Transfection

The lentiviral construct stubRFP‐sensGFP‐LC3 was purchased from GeneChem. Briefly, PC12 cells were transfected according to the manufacturer's protocol. After 16 h, the medium was replaced with fresh culture medium. Transfection efficiency was assessed under a confocal microscope at 72 h after transfection. Subsequently, stable cell lines were selected using puromycin‐containing medium and used for further experiments.

### Quantitative Real‐Time PCR

Total RNA was isolated from either spinal cord tissues of rats following SCI or treated PC12 cells using Trizol reagent, with the volume adjusted according to tissue weight (for spinal cord samples) or well size (for PC12 cells). RNA concentration and purity were determined using NanoDrop spectrophotometry. Subsequentially, RNA was reverse‐transcribed into cDNA using a reverse transcription kit (Accurate Biology, AG11728), followed by amplification with a SYBR Green qPCR kit (Accurate Biology, AG11739).

The primers used for amplification:

Pon3: 5’‐TGACTGTTGATCCAGCCACC‐3’(forward)
5’‐AATACAGTGTGCTCACCCGG‐3’ (reverse).
S1PR1: 5’‐TCTGGCTGTGCTGAACTCAG‐3’(forward)
5’‐CTGCGGCTAAATTCCATGCC‐3’ (reverse).
GAPDH: 5’‐TGACTGTTGATCCAGCCACC‐3’(forward)
5’‐AATACAGTGTGCTCACCCGG‐3’ (reverse).

### Methylation‐Specific PCR(MSP)

Briefly, following treatment, genomic DNA was extracted from PC12 cells using a genomic DNA extraction kit (Solarbio, D1700), and DNA concentration and purity were assessed by NanoDrop. The DNA was then converted and purified using a DNA bisulfite conversion kit (Beyotime, D0068S), followed by MSP using a MSP kit (TIANGEN, EM101). Finally, PCR products were analyzed by agarose gel electrophoresis.

The primers used for PCR amplification were designed using MethPrimer (https://methprimer.com/):

Pon3‐M: 5’‐TTTTTAAGTGGTTTTGAATTATCGG ‐3’ (forward)
5’‐CTACCTACACCTCCTAACAAATACG ‐3’ (reverse).
Pon3‐U: 5’‐TTTTTAAGTGGTTTTGAATTATTGG ‐3’ (forward)
5’‐ACCTACACCTCCTAACAAATACACT‐3’ (reverse).
M: methylated, U: unmethylated.

### Statistical Analysis

All data are presented as the mean ± standard error from triplicate experiments and have been normalized (n = 3). Between‐group comparisons were analyzed by two‐tailed unpaired t‐tests or one‐way/two‐way ANOVA, followed by Tukey's multiple comparisons test (SPSS 24.0 and GraphPad Prism 9.4.1). Statistical significance was defined as ns: p > 0.05; *p<0.05, **p<0.01, ***p<0.001, and ****p<0.0001.

## Conflict of Interest

The authors declare no conflict of interest.

## Author Contributions

B.P. conceived and designed the project with W.X. and L.Z. B.P. and H.L. analyzed the experimental data. B.P. wrote the first draft of the manuscript, and all the authors participated in the commentary and revision of the first draft and reviewed the final manuscript.

## Supporting information



Supporting Information

## Data Availability

The data that support the findings of this study are available from the corresponding author upon reasonable request.
