# Supplementary material for: Dnmt1 Alleviates S1PR1‐Mediated Pyroptosis after Spinal Cord Injury through Regulating Pon3 Expression
Source: Adv Sci (Weinh). 2025 Aug 30;12(42):e07330. doi: 10.1002/advs.202507330 (PMC12622493; doi:10.1002/advs.202507330)
Supplement: Supplementary file 1 — Supporting Information [file ADVS-12-e07330-s001.docx]

**Supporting Information**

Dnmt1 Alleviates S1PR1-Mediated Pyroptosis after Spinal Cord Injury through Regulating Pon3 Expression

Birong Peng, Haolong Lin, Mi Zhang, Wenhao Kuang, Jiaqi Zhang, Shuai Wang, Yuanfang Sun, Wenning Xu^*^, Lixin Zhu^*^

**1. Supplementary Figures**


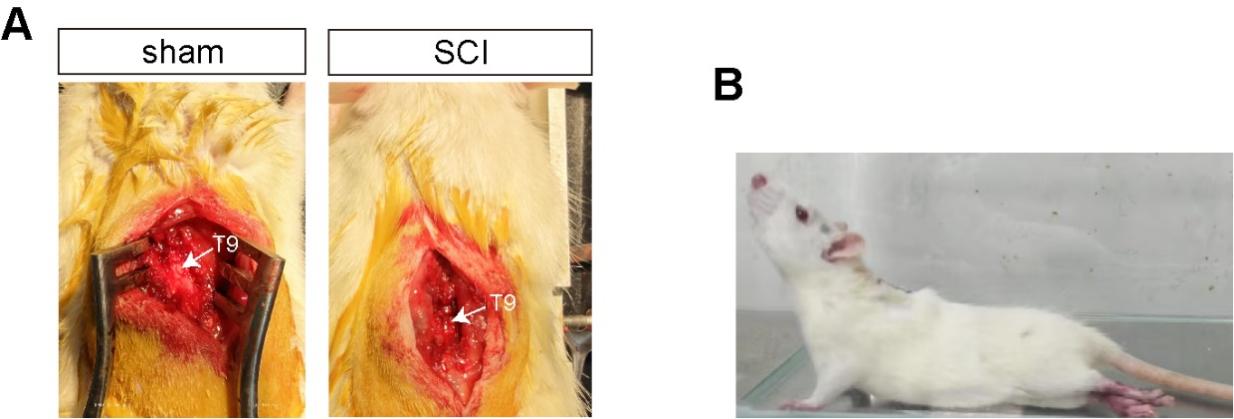


**Figure S1. Establishment of the rat SCI model.** (A) Dorsal spinal cord injury induced by impact (arrow). (B) Gross morphology of the rat at 1 day after SCI.


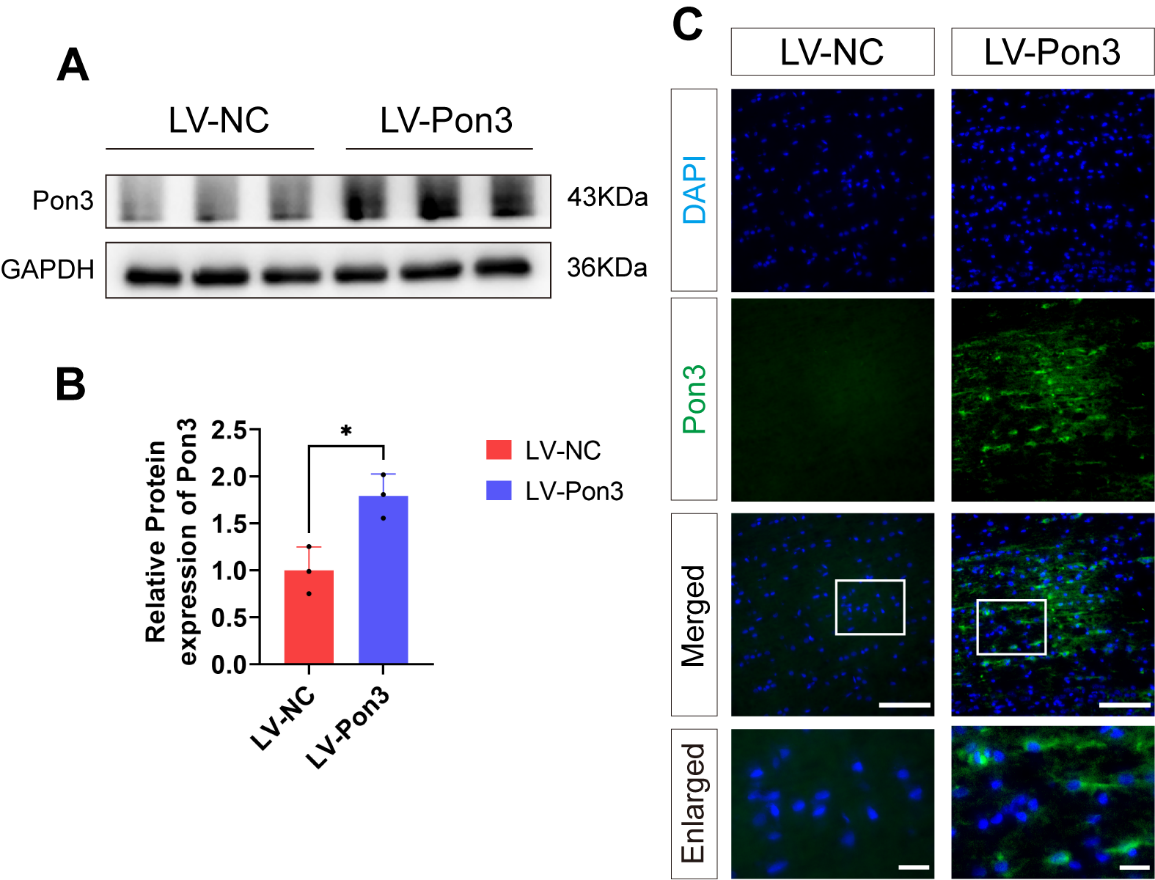


**Figure S2. Validation of Pon3 overexpression efficiency in rats.** (A) Western blot analysis of Pon3 protein expression in the spinal cord at 3 days after SCI. (B) Quantitative analysis of Pon3 protein levels after SCI (n = 3). (C) Double immunofluorescence staining for Pon3 (green) and DAPI (blue) in the spinal cord (scale bar: 100 µm in main panels; 20 µm in enlarged insets). Data are presented as mean ± SEM. Significance was determined by two-tailed unpaired t-tests. * p < 0.05.


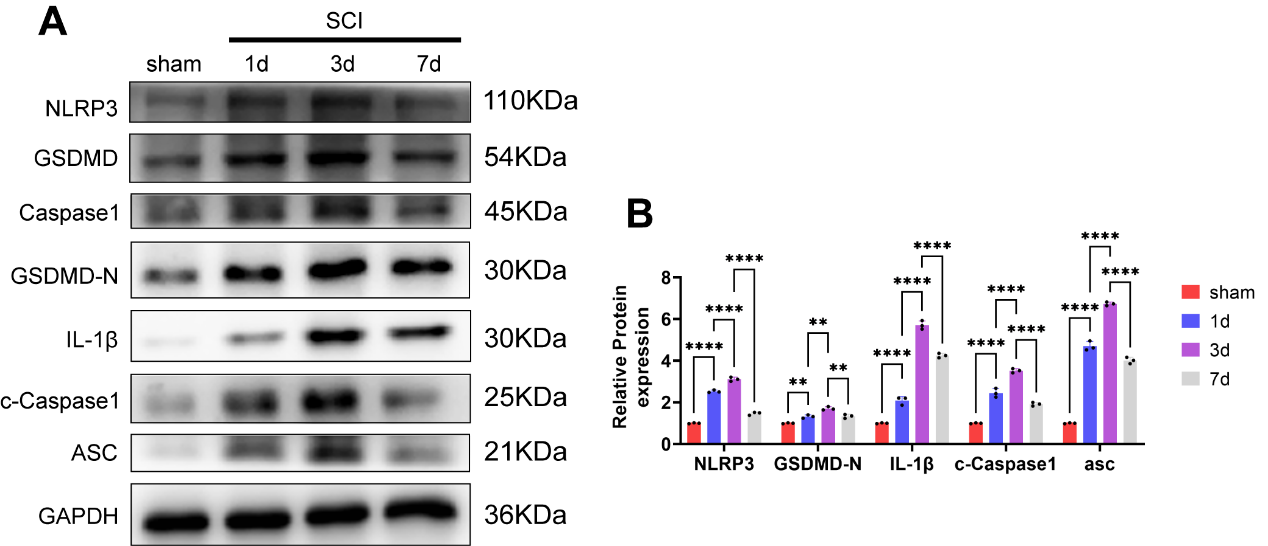


**Figure S3****. Temporal changes in pyroptosis after spinal cord injury (SCI).** (A-B) Western blot analysis of pyroptosis markers' expression levels in rats after SCI, with quantitative analysis (n = 3). Data are presented as mean ± SEM. Significance was determined by two-way ANOVA, followed by Tukey’s multiple comparisons test. ** p < 0.01; ****p <0.0001.


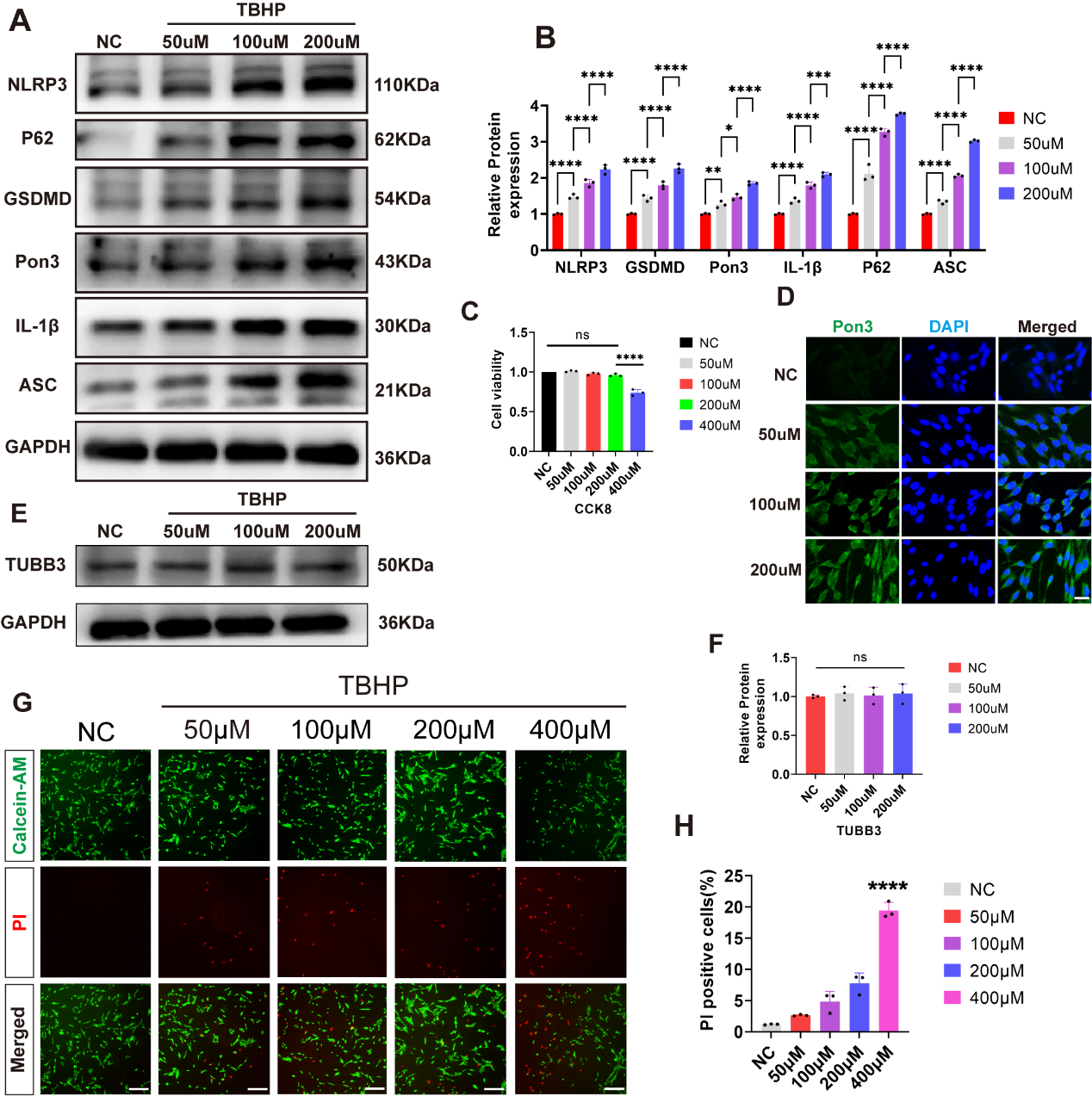


**Figure S4.** **TBHP induces pyroptosis and modulates Pon3 expression in PC12 cells.** (A-B) Western blot analysis of pyroptosis markers, Pon3, and autophagy substrate P62 protein expression levels in PC12 cells after TBHP stimulation, with quantitative analysis (n = 3). (C) Cell viability measured by CCK-8 assay in PC12 cells treated with 0-400 μM TBHP (n = 3). (D) Immunofluorescence staining of Pon3 (green) with DAPI (blue) in TBHP-stimulated PC12 cells (scale bar: 20 µm). (E-F) Western blot analysis of TUBB3 protein expression levels in PC12 cells after TBHP stimulation, with quantitative analysis (n = 3). (G-H) Live/dead cell staining to assess cell survival status and quantify dead cells in PC12 cells stimulated with TBHP (n = 3; scale bar: 200 µm). Data are presented as mean ± SEM. Significance was determined by one-way or two-way ANOVA, followed by Tukey’s multiple comparisons test. ns: p > 0.05; * p < 0.05; ** p < 0.01; *** p <0.001; ****p <0.0001.


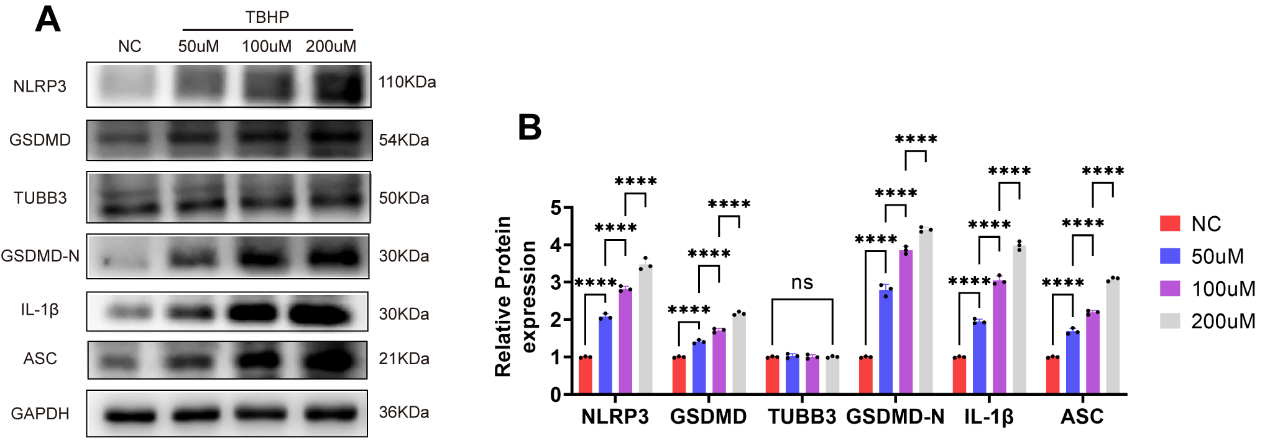


**Figure S5. TBHP induces pyroptosis in** **rat primary neurons.** (A-B) Western blot analysis of pyroptosis markers, and TUBB3 protein expression levels in rat primary neurons after TBHP stimulation, with quantitative analysis (n = 3). Data are presented as mean ± SEM. Significance was determined by two-way ANOVA, followed by Tukey’s multiple comparisons test. ns: p > 0.05; ****p <0.0001.


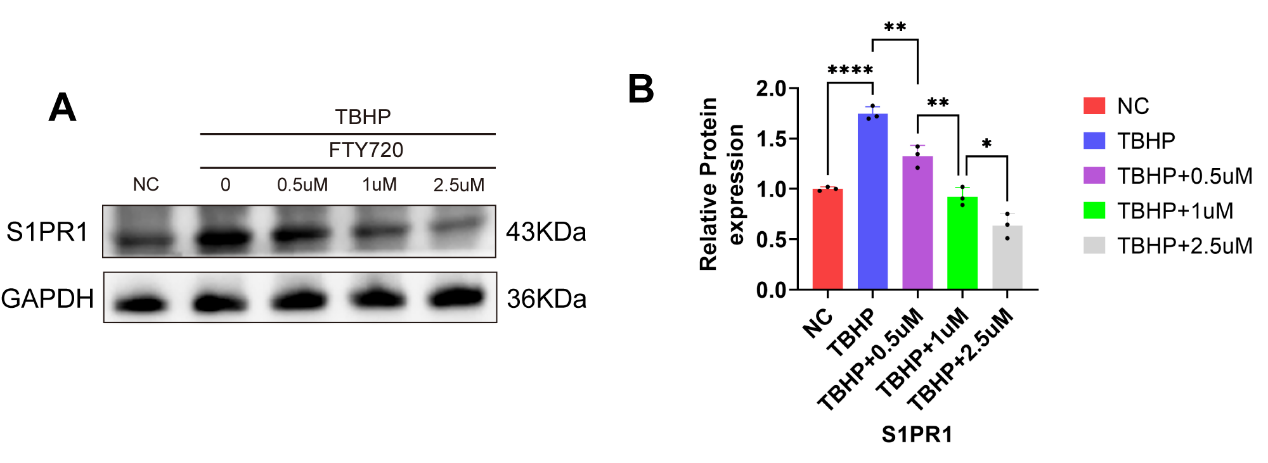


**Figure S6. Validation of the efficiency of FTY720 in inhibiting S1PR1 in PC12 cells.** (A-B) Western blot analysis of S1PR1 protein expression levels and quantitative results in PC12 cells treated with TBHP and FTY720 (n = 3). Data are presented as mean ± SEM. Significance was determined by one-way ANOVA, followed by Tukey’s multiple comparisons test. * p < 0.05; ** p < 0.01; ****p <0.0001.

**
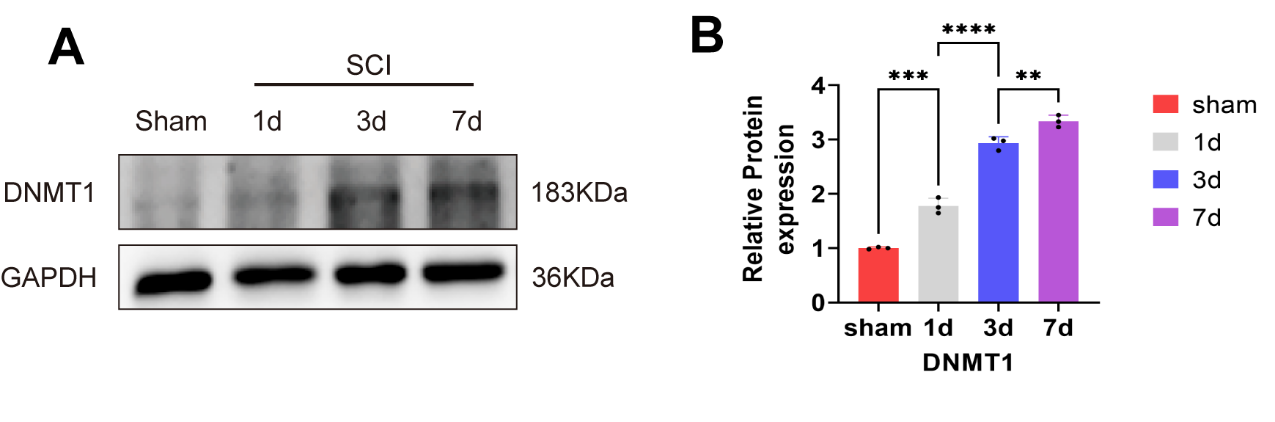
**

**Figure S7. Temporal changes of Dnmt1 expression after SCI.** (A-B) Western blot analysis and quantitative analysis of Dnmt1 protein expression in the spinal cord at different time points after SCI (n = 3). Data are presented as mean ± SEM. Significance was determined by one-way ANOVA, followed by Tukey’s multiple comparisons test. ** p < 0.01; *** p <0.001; ****p <0.0001.


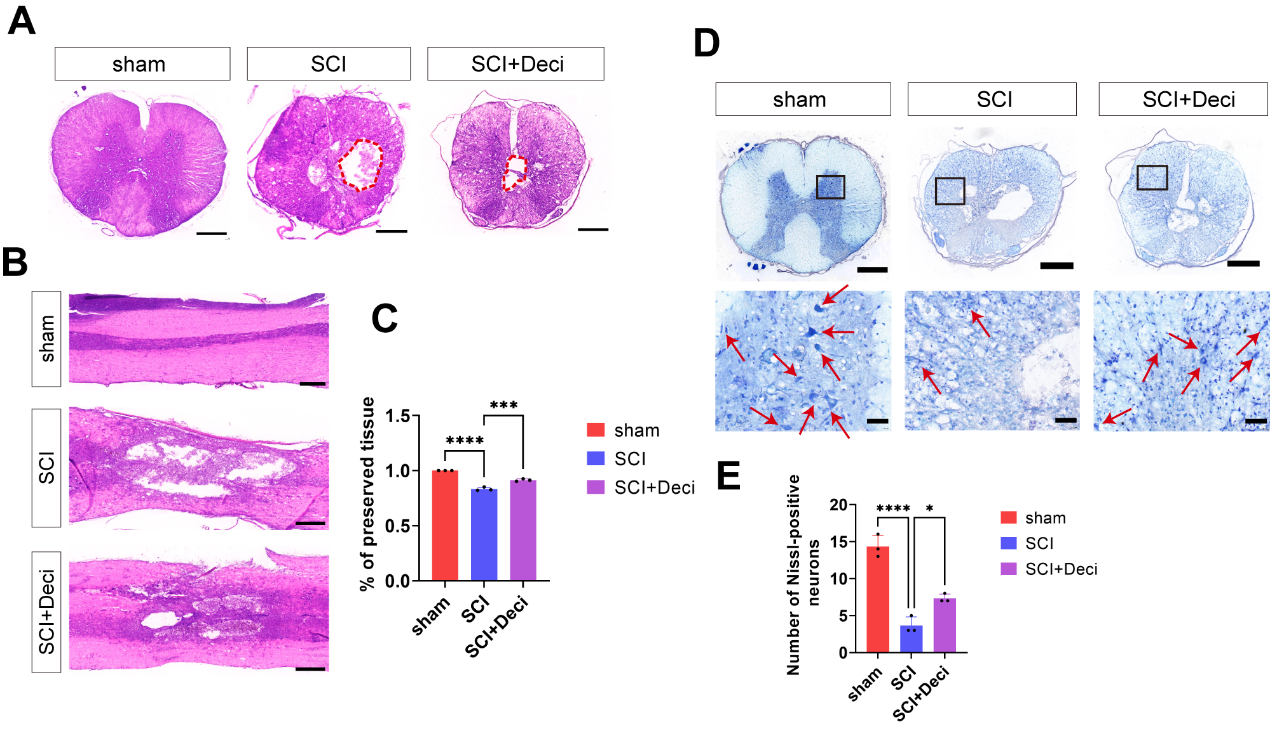


**Figure S8. Decitabine (Deci) treatment reduces tissue damage after spinal cord injury (SCI).** (A-C) Sagittal and transverse HE staining images at day 28 after SCI, and quantitative analysis of the percentage of relatively preserved spinal cord tissue (n = 3; scale bar: 500 µm). (D-E) Nissl staining images of spinal cord transverse sections at day 28 after SCI, and quantitative analysis of neurons (n = 3; Scale bars: 500 µm in main panels; 50 µm in enlarged insets). Data are presented as mean ± SEM. Significance was determined by one-way ANOVA, followed by Tukey’s multiple comparisons test. * p < 0.05; *** p <0.001; ****p <0.0001.


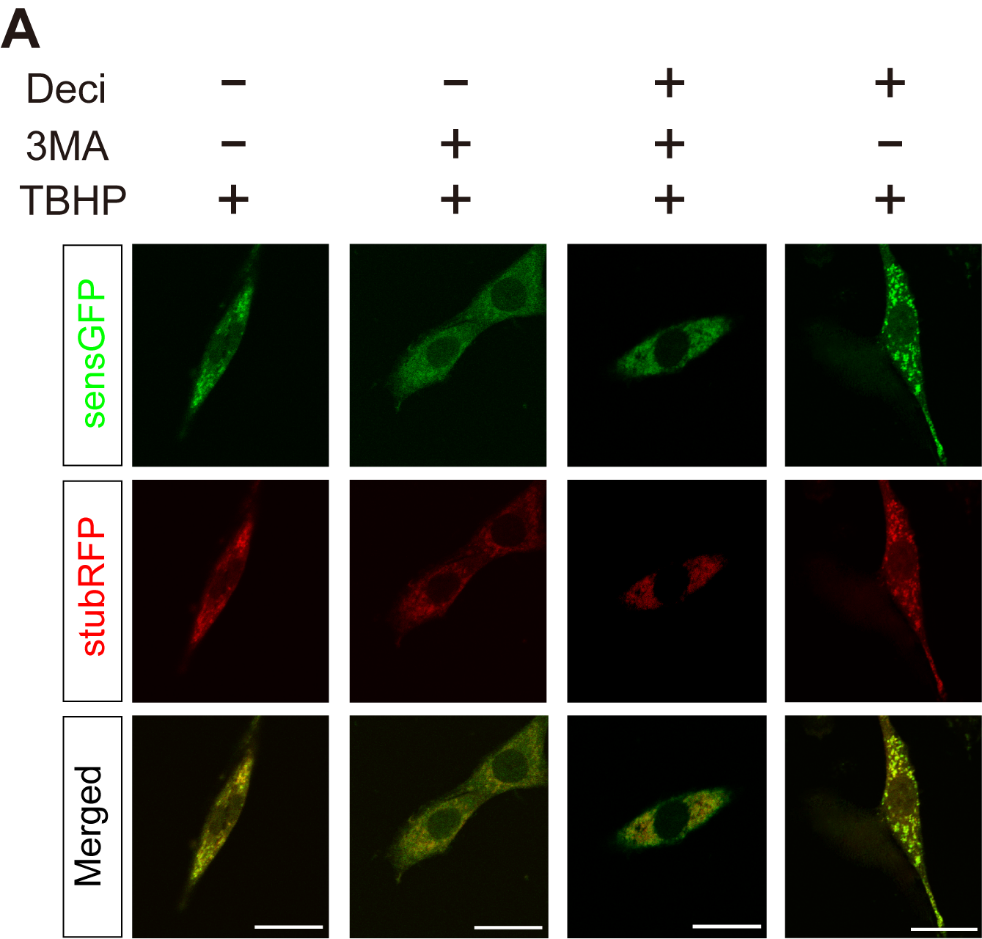


**Figure S9. Detection of Autophagic Flux Using Lentiviral stubRFP-sensGFP-LC3 Dual-Fluorescence System.** (A) Representative Images of Autophagic Flux in PC12 Cells Treated with 3-MA or Deci, Captured by Confocal Microscopy (scale bar: 20 µm).

**
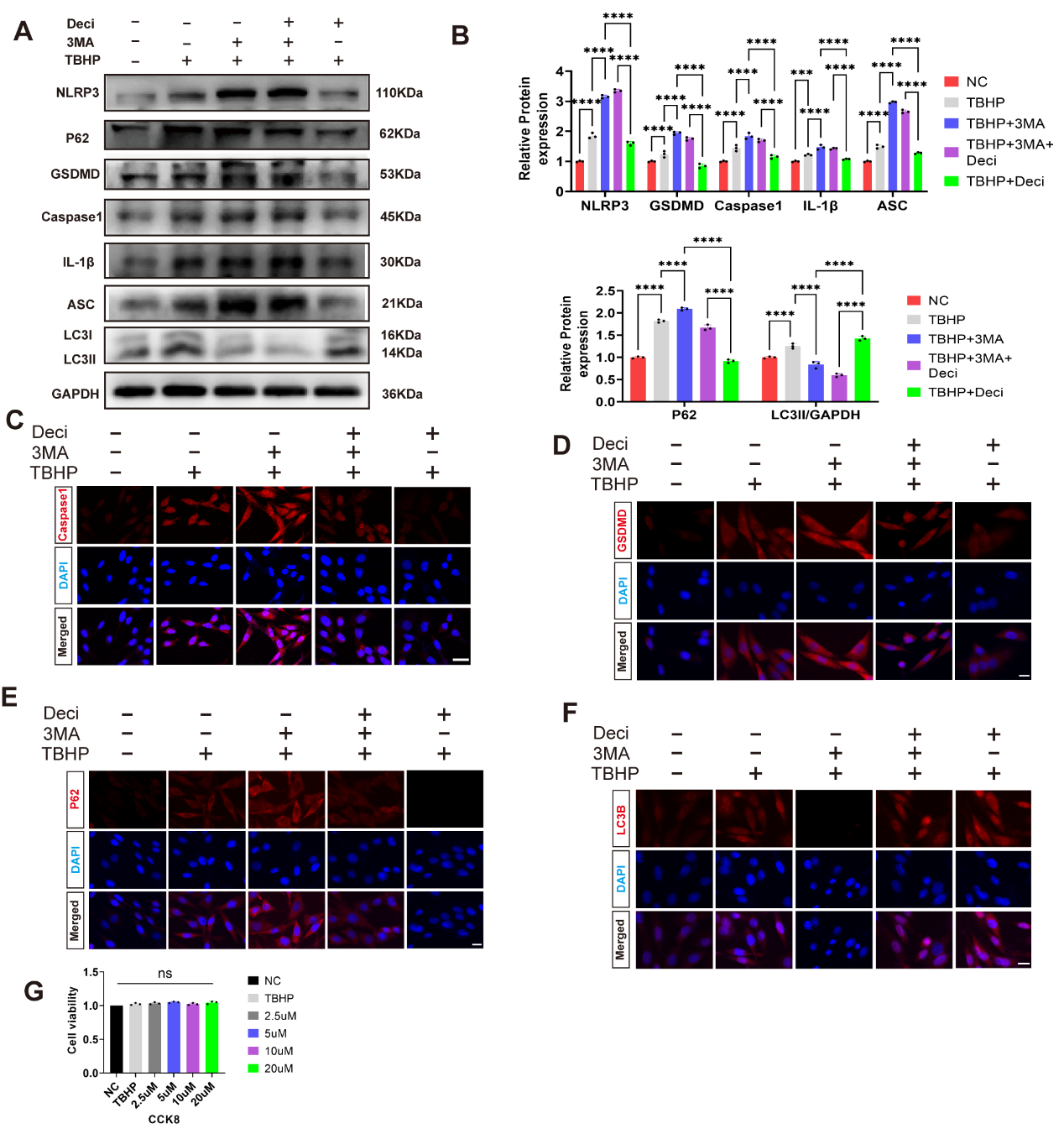
**

**Figure S10. Deci attenuates TBHP-induced pyroptosis in PC12 cells by enhancing autophagy.** (A-B) Western blot detection and quantitative analysis of protein expression levels of pyroptosis markers and autophagy markers (n = 3). (C-F) Immunofluorescence detection of the expression levels of Caspase1 (red), GSDMD (red), P62 (red), and LC3B (red) (scale bar: 20 µm). (G) CCK-8 Assay for Cell Viability of PC12 Cells Treated with Different Concentrations of Deci. Data are presented as mean ± SEM. Significance was determined by two-way ANOVA, followed by Tukey’s multiple comparisons test. ns: p > 0.05; ****p <0.0001.


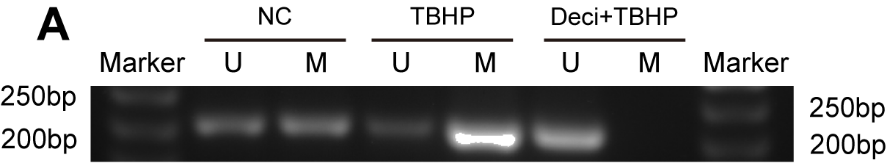


**Figure S11. Impact of Dnmt1 Knockdown on Pon3 Promoter Methylation Status in PC12 Cells.** (A) Methylation-Specific PCR (MSP) Analysis of Pon3 Promoter Methylation Levels (M: methylation-specific PCR products; U: unmethylated PCR products).
